# Shifting Milestones of Natural Sciences: The Ancient Egyptian Discovery of Algol’s Period Confirmed

**DOI:** 10.1371/journal.pone.0144140

**Published:** 2015-12-17

**Authors:** Lauri Jetsu, Sebastian Porceddu

**Affiliations:** 1 Department of Physics, P.O. Box 64, FI-00014 University of Helsinki, Finland; Hebrew University, ISRAEL

## Abstract

The Ancient Egyptians wrote Calendars of Lucky and Unlucky Days that assigned astronomically influenced prognoses for each day of the year. The best preserved of these calendars is the Cairo Calendar (hereafter CC) dated to 1244–1163 B.C. We have presented evidence that the 2.85 days period in the lucky prognoses of CC is equal to that of the eclipsing binary Algol during this historical era. We wanted to find out the vocabulary that represents Algol in the mythological texts of CC. Here we show that Algol was represented as Horus and thus signified both divinity and kingship. The texts describing the actions of Horus are consistent with the course of events witnessed by any naked eye observer of Algol. These descriptions support our claim that CC is the oldest preserved historical document of the discovery of a variable star. The period of the Moon, 29.6 days, has also been discovered in CC. We show that the actions of Seth were connected to this period, which also strongly regulated the times described as lucky for Heaven and for Earth. Now, for the first time, periodicity is discovered in the descriptions of the days in CC. Unlike many previous attempts to uncover the reasoning behind the myths of individual days, we discover the actual rules in the appearance and behaviour of deities during the whole year.

## Introduction

The Ancient Egyptians referred to celestial events indirectly [1–4] by relating them to mythological events. Many prognoses in the Calendars of Lucky and Unlucky Days have been connected to astronomical observations [1, 5–7]. Such connections between astronomical events and prognosis texts have been uncovered in most cases only for individual days [6, 8, 9]. The *P*_M_ = 29.6 days period of the *Moon* has been discovered in CC [10]. We have claimed that this document also contains the *P*_A_ = 2.85 days period of the eclipsing binary *Algol* [11]. However, it not a straightforward task to identify those indirect mythological references that are influenced by *Algol* in CC. Here we present a statistical analysis that reveals which CC prognosis texts describe *Algol*’s regular variability.

The Ancient Egyptian year contained 12 months (*M*) of 30 days (*D*) and five additional “epagomenal” days. CC gives three prognoses for each *D* of every *M* (G = “gut” = “good” and S = “schlecht” = “bad”) [11, 12]. CC also gives textual descriptions of the daily prognoses (S1 Fig). We study the dates of 28 selected words (hereafter SWs) in these mythological texts of CC. The dates are transformed into series of time points *t*_i_ with Eq (2). The *P*_A_ and *P*_M_ signals were originally discovered [11] from six large samples of lucky prognoses (*n* = 6 × 564 = 3384). We use these six samples to determine the zero epochs *t*_E_ of Eq (9) for the *P*_A_ and *P*_M_ signals. The time points leading to the discovery of these signals were close to phase, *ϕ* = 0, of Eq (5) using the ephemerides of Eqs (11) and (12) based on these zero epochs *t*_E_. The lucky prognoses of each SW are a subsample of the above mentioned large samples of lucky prognoses. We compute an impact parameter *z*_x_ for the *t*_i_ of each SW with Eq (10). The time points *t*_i_ of the lucky prognoses of any particular SW may strengthen (if *z*_x_ > 0) or weaken (if *z*_x_ < 0) the *P*_A_ and *P*_M_ signals. The impact parameter *z*_x_ is used for identifying the SWs having lucky prognoses close to phase, *ϕ* = 0, computed with the ephemerides of Eqs (11) and (12). We will show that *Algol* and the *Moon* were at their brightest close to phase *ϕ* = 0 with these two ephemerides. Hence, *Algol*’s eclipse and the New *Moon* occurred close to *ϕ* = 0.5.

Our statistical analysis also confirms two general things regarding the origin of the mythological texts of CC. First, the appearances and feasts of various deities are not independent of the prognoses, or randomly assigned, but regulated by the same periodic patterns. Second, the deities are used to represent the same astronomical phenomena that were also used to choose the prognoses for the days of the year.

## Materials

In this section, we transform the dates of 28 SWs in the mythological texts of CC into series of time points *t*_i_. Our main aim is that all stages of the production of these data can be replicated. With these instructions, similar series of time points can be produced for any particular SW in CC or other similar calendars, where the SW dates are available. We create the data in two stages: Identification of SW dates and Transformation of SW dates into series of time points.

### Identification of SW dates

CC is the best preserved Calendar of Lucky and unlucky Days. As in our two previous studies [10, 11], we use the best preserved continuous calendar found on pages recto III-XXX and verso I-IX of papyrus Cairo 86637. There are two CC translations, in English by Bakir [13] and in German by Leitz [12]. Our SWs have been identified according to the hieroglyphic transcription in Leitz [12] and the two aforementioned translations. In case of discrepancy we have consulted the photocopies of the original hieratic text given by Leitz [12]. For the sake of convenience, we quote sentences according to Bakir’s English translation despite its imperfections because there is neither space nor reason to discuss the linguistic details of the text in the present article. This approach should ascertain that our study of the CC sentences is objective. In other words, we do not ourself translate any CC sentences into English, but we do check which individual Ancient Egyptian SWs were also identified by Bakir [13] and Leitz [12]. There is only one exception to our sentence quotation rule, i.e. the CC text connected to *Horus* where Bakir [13] did not identify *Horus*, but Leitz [12] and we did (Algol in lucky prognoses: the text at date *g*_i_(1, 10)).

Naturally, we can not analyse all words in CC. Our main selection criterion is to include deities, nouns or locations that could have been used to indirectly describe periodic phenomena, due to their significant mythological properties and multiple occurrences in the text. Our list of SWs is not absolute and we give all the necessary information for other researchers to repeat our experiment on other SWs we may have ignored. Our 28 SWs in Ancient Egyptian language are given in Table 1.

**Table 1:** List of SWs in Ancient Egyptian language.

*ꜣbdw*	*Abydos*	*ḥm*	*Majesty*
*ddw*	*Busiris*	*rmt*	*Man* (mankind, men)
*tꜣ*	*Earth* (ground, land)	*nwt*	*Nut*
*ḫfty*	*Enemy*	*nnw*	*Nun* (primeval waters)
*psdt*	*Ennead*	*wnn-nfr*	*Onnophris*
*i͗rt*	*Eye*	*wsi͗r*	*Osiris*
*ḫt*	*Fire*	*rꜥ*	*Re* (the Sun)
*nsrt*	*Flame*	*sbi͗*	*Rebel* (to rebel)
*i͗myw-ḫt*	*Followers* (following)	*sḫmt*	*Sakhmet*
*i͗b*	*Heart*	*sth*	*Seth*
*pt*	*Heaven* (sky)	*šw*	*Shu* (sunlight)
*i͗wnw*	*Heliopolis*	*sbk*	*Sobek*
*ḥr*	*Horus*	*dḥwty*	*Thoth*
*mꜣi͗*	*Lion*	*wdꜣt*	*Wedjat*

We do not use the occurrences of our SWs in compound words and composite deities (e.g. House of Horus or Ra-Horakhti), because it is uncertain to which word, if not both, the prognosis is connected to. Our identifications of 28 SWs in CC are given in Table 2. It shows that all our 460 SW date identifications are the same as those made by Leitz [12] (Column 5: 460× “Yes”). However, 21 of our identifications were not made by Bakir [13] (Column 6: 21× “No”: 1× “Earth”, 2× “Enemy”, 4× “Fire”, 12× “Heart”, 1× “Horus” and 1× “Osiris”). Fortunately, most days have combinations “GGG” or “SSS” and we know that the lucky or unlucky SW prognosis is certainly correct. We ignore the heterogeneous combinations “HET” (like “SSG”at *D* = 6 and *M* = 1), because the correct SW prognosis is uncertain. The dates with an unknown prognosis combination, “- - -”, are naturally also ignored. Our notations for the number of lucky and unlucky dates for each SW are *n*_G_ and *n*_S_. For example, “Abydos” has *n*_G_ = 3 and *n*_S_ = 2.

**Table 2:** SWs identified in CC.

SW	*D*	*M*	Prog	Ltz	Bkr
Abydos	13	3	SSS	Yes	Yes
Abydos	17	3	- - -	Yes	Yes
Abydos	11	4	GGG	Yes	Yes
Abydos	18	5	GGG	Yes	Yes
Abydos	27	6	- - -	Yes	Yes
Abydos	28	7	GGG	Yes	Yes
Abydos	13	8	SSS	Yes	Yes
Abydos	23	8	- - -	Yes	Yes
Busiris	26	2	SSS	Yes	Yes
Busiris	14	5	SSS	Yes	Yes
Busiris	26	5	SSS	Yes	Yes

The selected word (SW) identified on day (*D*) of month (*M*) in CC. The daily prognosis combinations (Prog) are “GGG” (All lucky), “SSS” (All unlucky), “- - -” (All unknown) or “HET” (Heterogeneous). The same SW was identified at the same date by Leitz [12] (Ltz = ”Yes” or “No”) and by Bakir [13] (Bkr = ”Yes” or “No”). The twelve first lines of all 460 lines are shown here for guidance regarding the contents of this ASCII file which can be downloaded on Dryad (http://dx.doi.org/10.5061/dryad.tj4qg).

### Transformation of SW dates into series of time points

The dating of CC does not influence the results of our currect analysis, because we transform the time points to unit vectors with Eq (6). The mutual directions between these unit vectors do not depend on the chosen zero epoch *t*_0_ in time. Adding any positive or negative constant value to these time points rotates all unit vectors with the same constant angle. Hence, our significance estimates of Eqs (8) and (13) do not depend on the connection between Gregorian and Egyptian days. The only assumption made in our Eq (2) below is that the separation between two subsequent days is exactly one day during the particular year that CC happens to describe.

The transformation relations in Eqs (2) and (3) of Jetsu et al.[11] were

$$\begin{array}{c} t_{i}=N_{E}-1+a_{i}, \end{array}$$ (1)

where *N*_E_ = 30(*M* − 1) + *D* and *a*_i_ was a decimal part. This decimal part *a*_i_ was different for each of the three parts of the day. The *a*_i_ values depended on the chosen transformation between Egyptian and Gregorian year, and on the chosen day division. The *P*_A_ and *P*_M_ signals were discovered in samples of series of time points SSTP = 1, 3, 5, 7, 9 and 11 in Jetsu et al.[11]. The size of each sample was *n* = 564. The period analysis results were the same for all these six samples, although their *a*_i_ values were different for every *N*_E_. The time points *t*_i_ of these six samples are given in Table 3.

**Table 3:** The time points *t*_i_ of lucky prognoses in Jetsu et al.[11].

SSTP = 1	SSTP = 3	SSTP = 5	SSTP = 7	SSTP = 9	SSTP = 11
0.080	0.095	0.076	0.120	0.142	0.114
0.239	0.284	0.227	0.359	0.426	0.341
0.399	0.473	0.379	0.739	0.784	0.727
1.080	1.095	1.076	1.120	1.142	1.113
1.240	1.284	1.227	1.360	1.425	1.340

The *t*_i_ values of SSTP = 1, 3, 5, 7, 9 and 11 from Table 3 in Jetsu et al. [11]. The five first lines of all 534 lines are shown here for guidance regarding the contents of this ASCII file which can be downloaded on Dryad (http://dx.doi.org/10.5061/dryad.tj4qg).

The mean of the decimal parts *a*_i_ of all these *n* = 6 × 564 = 3384 values of *t*_i_ is *m*_t_ = 0.33. In this study, the time point for an SW at the day *D* of the month *M* in CC is therefore computed from

$$\begin{array}{c} t_{i}=t_{i}\left(D,M\right)=N_{E}-1+m_{t}. \end{array}$$ (2)

This accuracy is sufficient, because we do not know to which part or parts of the day each SW refers to (*σ*_*t*_ ≈ ±0.^d^5) and some prognosis texts may refer to the previous or the next day (*σ*_*t*_ ≈ ±1.^d^5). The *t*_i_ of Table 3 (*n* = 6 × 564 = 3384) are also later used to determine the zero epochs *t*_E_ for the ephemerides connected to the *P*_A_ and *P*_M_ signals (Eqs (11) and (12)). Our “synchronization” of time points of Eqs (1) and (2) ensures that these ephemerides enable us to identify the SWs connected to the *P*_A_ and *P*_M_ signals. For a given *t* value, the inverse transformation is

$$\begin{array}{c} M=\text{INT}[\left(t+1-m_{t}\right)/30]+1 \end{array}$$ (3)

$$\begin{array}{c} D=t-m_{t}+1-30\left(M-1\right), \end{array}$$ (4)

where *INT* removes the decimal part of (*t* + 1 − *m*_t_)/30. In other words, if the analysis our data gives any particular *t* value, the *D* and *M* values of this *t* can be solved from Eqs (3) and (4).

The time points *t*_i_ for all dates with a “GGG” or “SSS” prognosis combination in CC are given in Table 4. These *t*_i_ are needed in computing the binomial distribution probabilities *Q*_B_ of Eq (13).

**Table 4:** The time points *t*_i_ of all GGG and SSS dates in CC.

*D*	*M*	*t*_i_	Prog
1	1	0.33	GGG
2	1	1.33	GGG
5	1	4.33	GGG
7	1	6.33	GGG
9	1	8.33	GGG
10	1	9.33	GGG
11	1	10.33	SSS
12	1	11.33	SSS
16	1	15.33	SSS
17	1	16.33	SSS

The day (*D*) and month (*M*) values in CC used in computing the time points (*t*_i_) for the days with the prognosis (Prog) combinations “GGG” or “SSS”. There are *N*_G_ = 177 and *N*_S_ = 105 days with a “GGG” and “SSS” combination, respectively. These data are from Table 1 in Jetsu et al. 2013 [11]. The ten first lines of all 282 lines are shown here for guidance regarding the contents of this ASCII file which can be downloaded on Dryad (http://dx.doi.org/10.5061/dryad.tj4qg).

## Methods

Let us assume that time is a straight line, where events are equidistant dots with a separation of 2*π*. If this line is wound on a *d* = 1 diameter wheel, the dots line up at the same point on the wheel. Removing some dots produces gaps in the time line, but the remaining dots will still line up on the wheel. However, they will not line up on a *d* ≠ 1 diameter wheel. This is an analogy for the Rayleigh test. It projects time points on a unit circle with the tested period *P*. These points line up in the same direction, if their time distribution is regular with the tested *P*.

### Analysis

If the Rayleigh method discovers the period *P* in a series of time points points **t** = [*t*_1_, *t*_2_, …, *t*_*n*_], it is possible to identify those subsamples t* of n* time points that strengthen this signal. In other words, the signal can be separated from noise. The phases of the *n* time points *t*_i_ are

$$\begin{array}{c} \phi_{i}=\text{FRAC}\left[\left(t_{i}-t_{0}\right)/P\right], \end{array}$$ (5)

where *t*_0_ is an arbitrary zero epoch and *FRAC* removes the integer part of (*t*_i_ − *t*_0_)/*P*. The unit vectors are

$$\begin{array}{c} r_{i}=\left[\cos\Theta_{i},\sin\Theta_{i}\right], \end{array}$$ (6)

where Θ_i_ = 360°, *ϕ*_i_ are the phase angles. The test statistic of the Rayleigh test is

$$\begin{array}{c} z={\left|R\right|}^{2}/n, \end{array}$$ (7)

where vector $R=\sum_{i=1}^{n}r_{i}$ points to Θ_R_ = atan(*R*_y_/*R*_x_), $R_{x}=\sum_{i=1}^{n}\cos\Theta_{i}$ and $R_{y}=\sum_{i=1}^{n}\sin\Theta_{i}$. The corresponding phase is *ϕ*_R_ = Θ_R_/(360°). Coinciding directions Θ_i_ give |**R**| = *n*, while random Θ_i_ give |**R**| ≈ 0. The critical level (i.e. significance) of the Rayleigh test is

$$\begin{array}{c} Q_{z}=e^{-z}. \end{array}$$ (8)

We use the ephemeris zero epoch

$$\begin{array}{c} t_{E}=t_{0}+P\phi_{R}. \end{array}$$ (9)

The mutual directions of **r**_i_ and the length |**R**| are invariant for any constant shift of *m*_t_, *t*_i_, *t*_0_ or *t*_E_. Using the above *t*_E_ of Eq (9), vector **R** points to Θ = Θ_R_ = 0°. All **r**_i_ with −90° < Θ_i_ < 90° strengthen the *P* signal, while the remaining **r**_i_ weaken it. The test statistic can be divided into $z=R_{x}^{2}/n+R_{y}^{2}/n$. We fix *t*_0_ = *t*_E_ in Eq (5) and compute the “impact” of any subsample t* on the *P* signal from

$$\begin{array}{c} z_{x}=(R_{x}/|R_{x}\left|\right)\left(R_{x}^{2}/n\right), \end{array}$$ (10)

where *R*_x_ is computed only for the *n* = n* time points of t*. These t* may strengthen (*z*_x_ > 0) or weaken (*z*_x_ < 0) the *P* signal, or represent noise (*z*_x_ ≈ 0).

Using the zero epoch *t*_0_ = 0 for the *n* = 6 × 564 time points *t*_i_ of the G prognoses in Table 3 gives the *t*_E_ values of Table 5 for the *P*_A_ and *P*_M_ signals with Eq (9).

**Table 5:** Values of *t*_E_ of the six samples.

*P*	SSTP = 1	SSTP = 3	SSTP = 5	SSTP = 7	SSTP = 9	SSTP = 11
2.85	0.45	0.45	0.44	0.61	0.61	0.60
29.6	3.42	3.42	3.42	3.58	3.58	3.58

These six large samples have *t*_E_ = 0.53 ± 0.09 for *P*_A_ and *t*_E_ = 3.50 ± 0.09 for *P*_M_. Hence, we use the following two ephemerides

$$\begin{array}{c} t_{0}=t_{E}=0.53,P=P_{A}=2.85\;\;\;(\text{Algol}) \end{array}$$ (11)

$$\begin{array}{c} t_{0}=t_{E}=3.50,P=P_{M}=29.6\;\;\;(\text{Moon}). \end{array}$$ (12)

for computing the phases *ϕ*_i_ of Eq (5). The lucky “GGG” prognoses of every SW are a subsample of the above six large samples of all “G” prognoses. We give the *z* and *z*_x_ values of Eqs (7) and (8) for any particular SW, if the analysed *t*_i_ of this SW reach *Q*_z_ ≤ 0.2 with the ephemerides of Eqs (11) or (12). These periodicities are called weak if 0.05 < *Q*_z_ ≤ 0.2.

**Fig 1:**
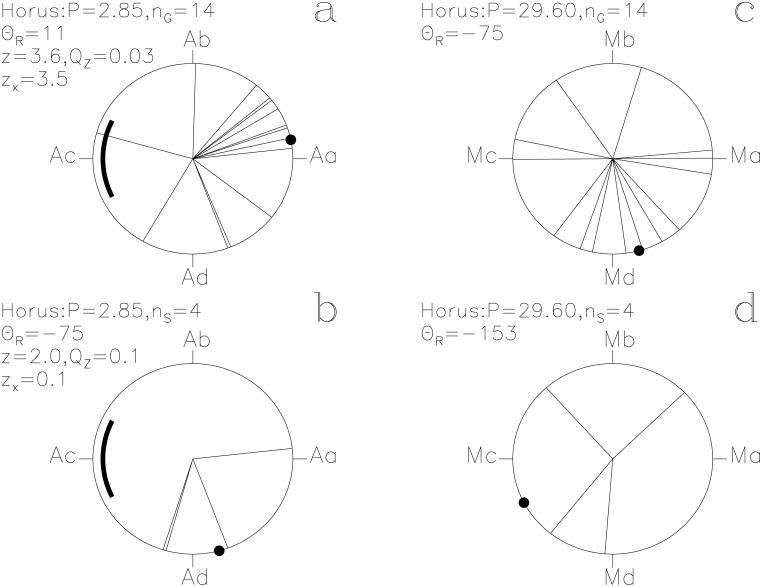
Horus. Time runs in the counter clock–wise direction on these unit circles. We give the *z*, *Q*_z_ and *z*_x_ values only when *Q*_z_ ≤ 0.2. The large black point indicates the Θ_R_ direction. (a) **g**_i_ with Eq (11). Point Aa is at *ϕ* = 0 ≡ 0°. The thick line centered on point Ac at *ϕ* = 0.5 ≡ 180° outlines the proposed phase for the 10 hr eclipse of Algol. (b) **s**_i_ with Eq (11). (c) **g**_i_ with Eq (12). Point Ma at *ϕ* = 0 ≡ 0° is close to the proposed Full *Moon* phase. (d) **s**_i_ with Eq (12)

**Fig 2:**
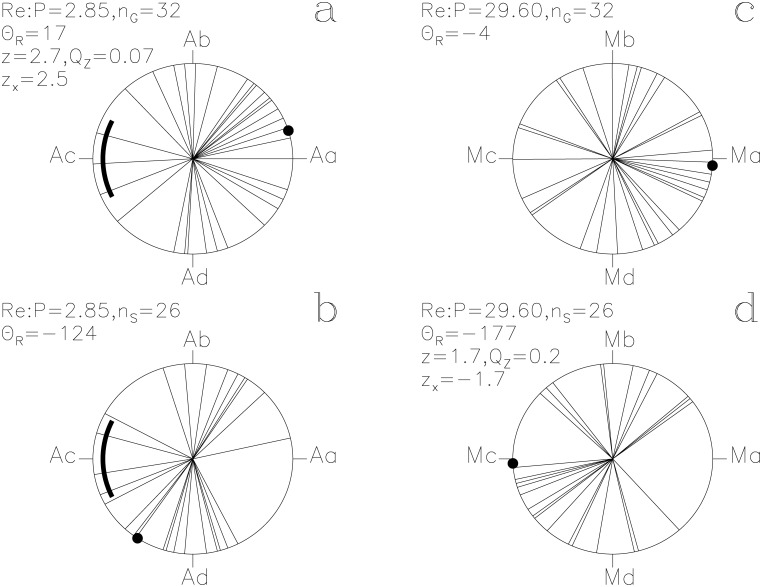
Re. otherwise as in Fig 1

**Fig 3:**
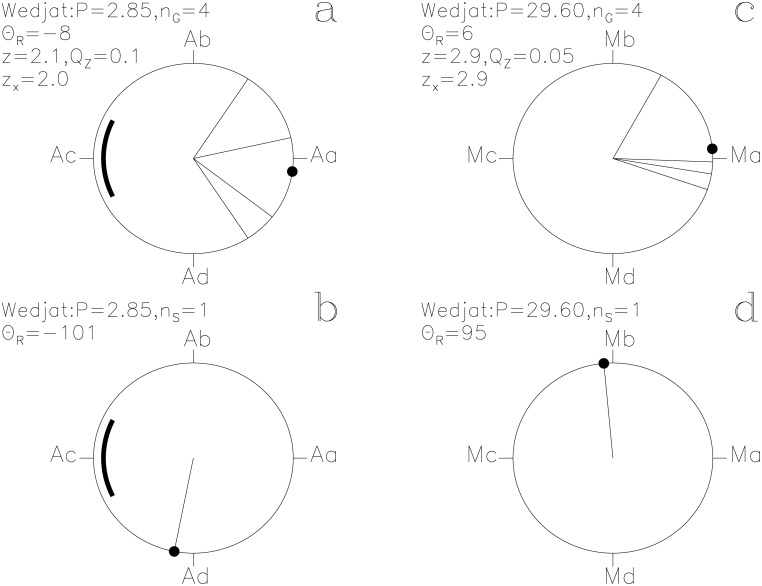
Wedjat. otherwise as in Fig 1

**Fig 4:**
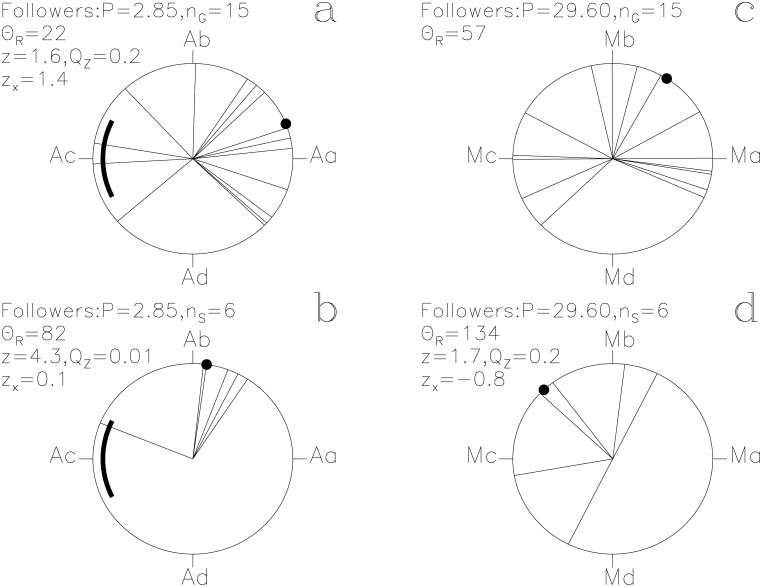
Followers. otherwise as in Fig 1

**Fig 5:**
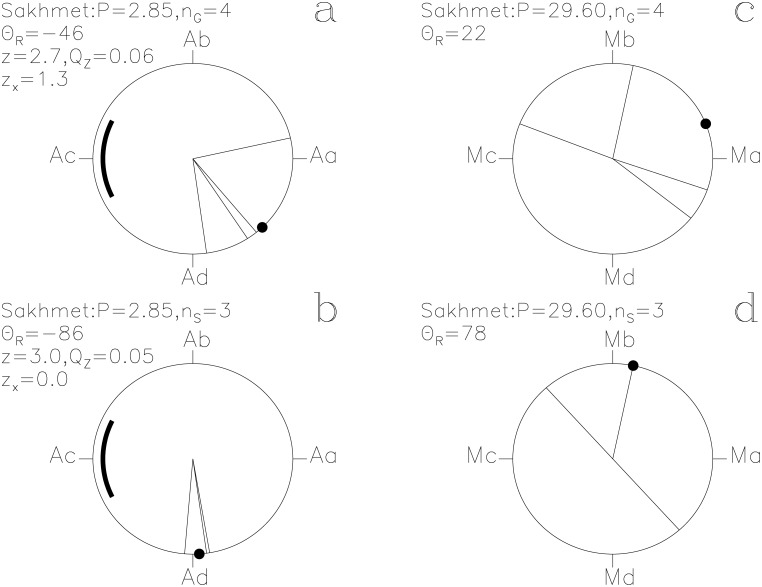
Sakhmet. otherwise as in Fig 1

**Fig 6:**
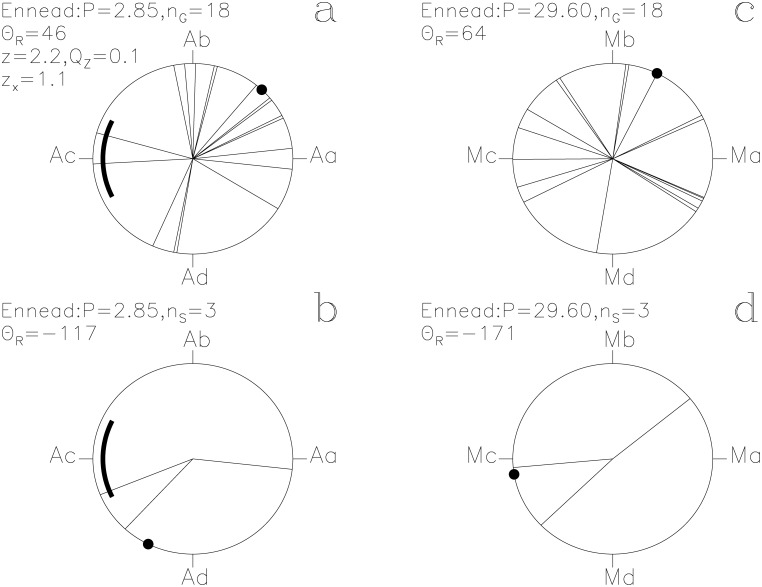
Ennead. otherwise as in Fig 1

**Fig 7:**
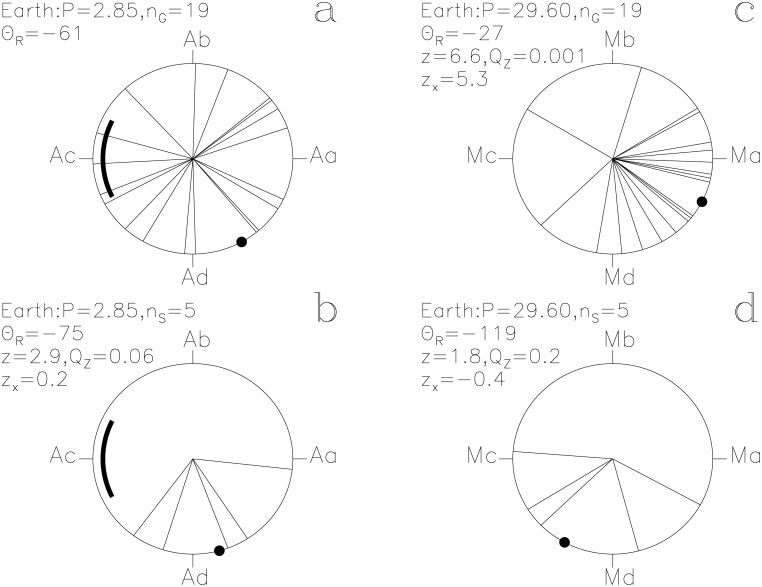
Earth. otherwise as in Fig 1

**Fig 8:**
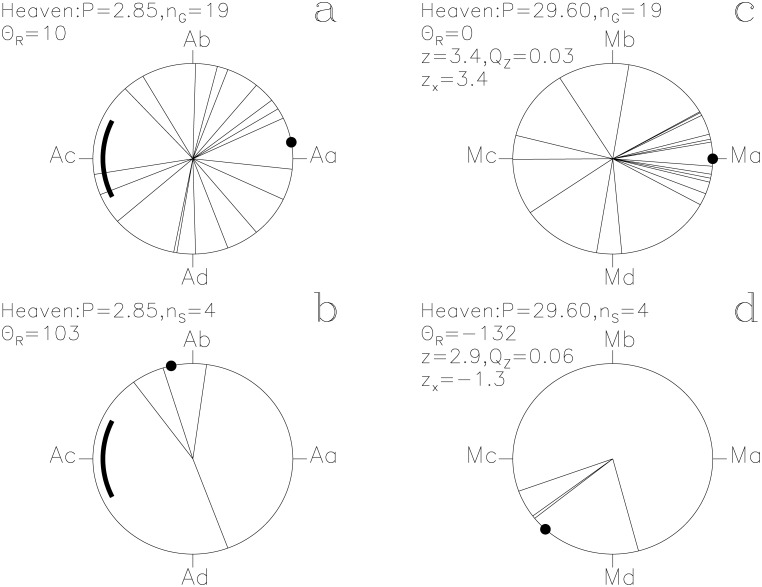
Heaven. otherwise as in Fig 1

**Fig 9:**
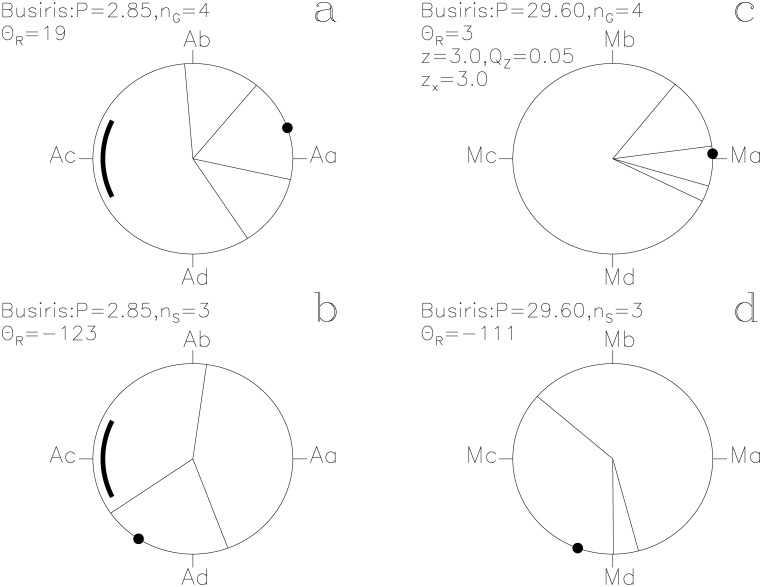
Busiris. otherwise as in Fig 1

**Fig 10:**
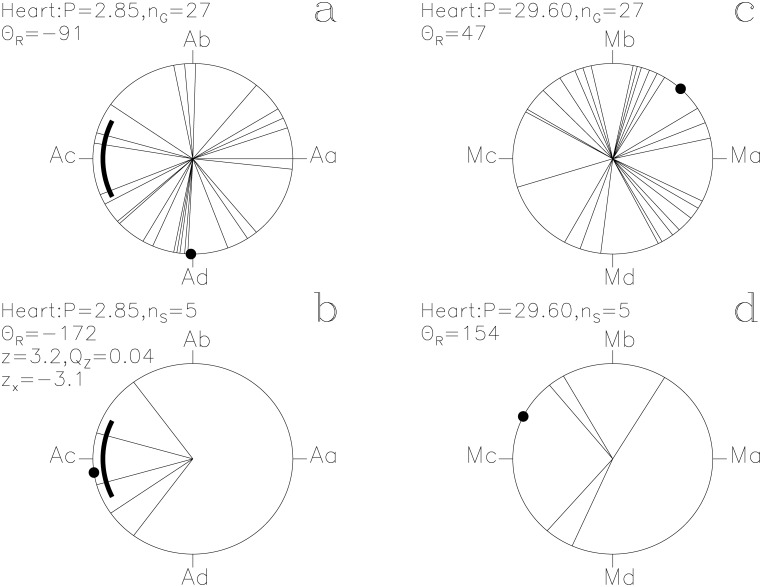
Heart. otherwise as in Fig 1

**Fig 11:**
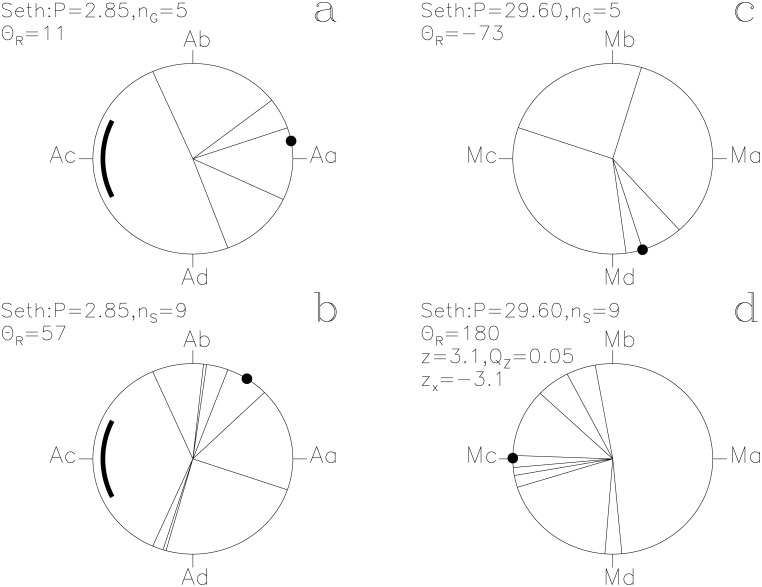
Seth. otherwise as in Fig 1

**Fig 12:**
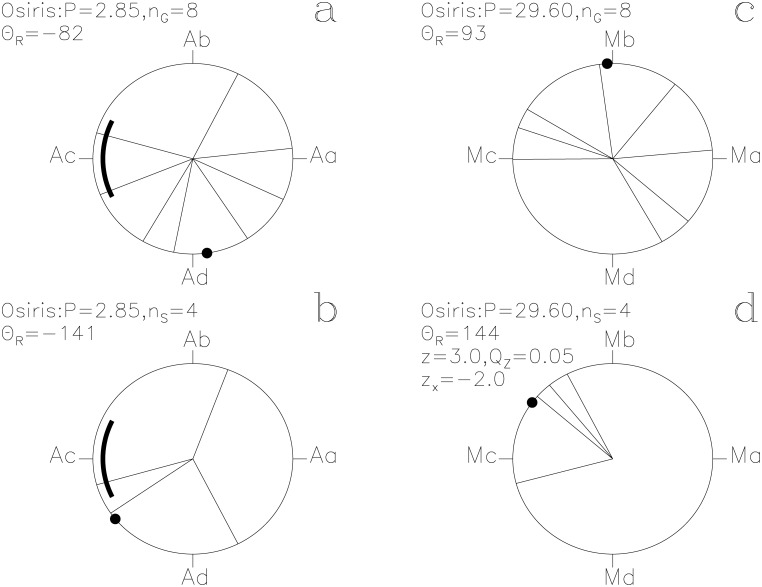
Osiris. otherwise as in Fig 1

**Fig 13:**
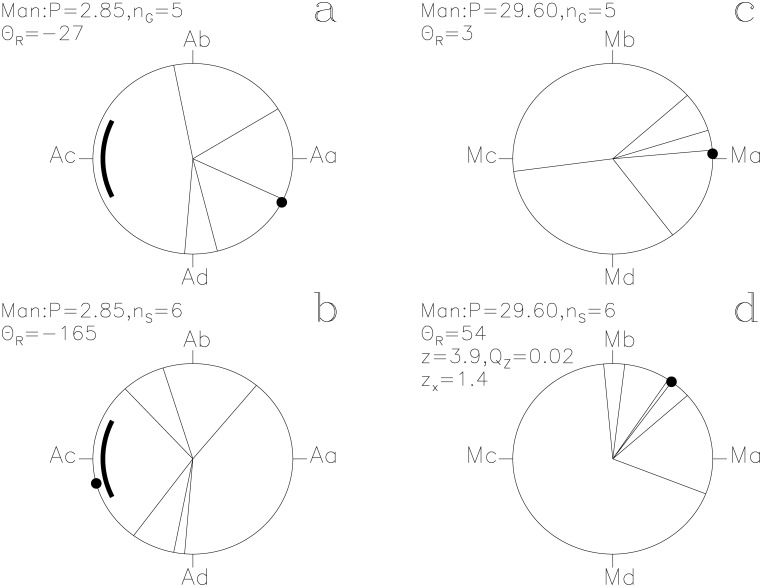
Man. otherwise as in Fig 1

In our Figs 1–13, we project the *t*_i_ of each SW to **r**_*i*_ = [cos Θ_i_, sin Θ_i_] on a unit circle, where time runs in the counter clock–wise direction. For the *P*_A_ signal, we define four points Aa, Ab, Ac and Ad. The first one, Aa, is at *ϕ* = 0 ≡ 0° with the ephemeris of Eq (11). The next three points Ab, Ac and Ad are separated by Δ*ϕ* = 0.25 ≡ 90°. Vectors **r**_i_ pointing between Ad ≡ −90° and Ab ≡ +90° give *z*_x_ > 0 and strengthen *P*_A_ signal, the other ones weaken it. Because *P*_A_ equals 57^d^/20, the *ϕ*_i_ of *t*_i_ separated by multiples of 57 days are equal. For clarity, we shift such overlapping *ϕ*_i_ values by Δ*ϕ* = 0.005 away from each other in our Figs 1–13. However, there are no such shifts in our computations. Our unambiguous terminology is:

*“Connected to the P_A_ signal”* ≡ *t*_i_ of an SW strengthen the *P*_A_ signal ≡ *z*_x_ ≥ 1.0 and *Q*_z_ ≤ 0.2 with the ephemeris of Eq (11).

*“Connected to Algol”* ≡ *t*_i_ of an SW show periodicity with *P*_A_, but their contribution to the *P*_A_ signal is insignificant when 0 ≤ *z*_x_ < 1.0 or they weaken this signal when *z*_x_ < 0 ≡ *z*_x_ < 1.0 and *Q*_z_ ≤ 0.2 with the ephemeris of Eq (11).

We use similar terminology for the *Moon* (Eq (12)), and Ma–Md points similar to Aa–Ad.

Our notations for the lucky and unlucky time points *t*_i_ of each SW are *g*_i_ and *s*_i_. The notations for their unit vectors **r**_i_ of Eq (6) are **g**_i_ and **s**_i_, respectively. The critical level *Q*_z_ measures the probability for the concentration of *all* *n*_G_ and *n*_S_ directions of **g**_i_ and **s**_i_ of each SW. These directions are embedded within the directions of *all* **g**_i_ (Table 4: *N*_G_ = 177) and **s**_i_ (Table 4: *N*_S_ = 105). We first choose the direction Θ_R_ of **R** for some SW. Then we identify the *n*_1_ directions of **g**_i_ or **s**_i_ of this SW that are among the *n*_2_ of all *N*_G_ or *N*_S_ directions closest to Θ_R_. For each SW, this gives the binomial distribution probability

$$\begin{array}{c} Q_{\text{B}}=P\left(n_{1},n_{2},N\right)=\overset{n_{2}}{\underset{i=n_{1}}{\sum }}\left(\begin{array}{c} n_{2}\\ i \end{array}\right)q_{\text{B}}^{i}{\left(1-q_{\text{B}}\right)}^{n_{2}-i}, \end{array}$$ (13)

where *N* = *N*_G_ or *N*_S_, and *q*_B_ = *n*_G_/*N*_G_ or *n*_S_/*N*_S_. This *Q*_B_ is the probability for that the directions of a particular SW occur *n*_1_ times, or more, among all *n*_2_ directions closest to Θ_R_. Many *Q*_z_ estimates based on small samples (*n*_G_ or *n*_S_) are unreliable, but the *Q*_B_ estimates based on large samples (Table 4: *N*_G_ = 177 or *N*_S_ = 105) are not.

All results of our analysis are given in S1 Table, where the results mentioned in text are marked with bold letters. The structure of S1 Table resembles the four panel structure of Figs 1–13. We give four separate tables for each SW. The results for the lucky and unlucky prognoses with *P*_A_ are those shown in figure panels “a” and “b”. The corresponding results for *P*_M_ are shown in figure panels “c” and “d”.

## Results

### Algol in lucky prognoses

Of all 28 SWs, only the lucky prognoses of *Horus*, *Re*, *Wedjat*, *Followers*, *Sakhmet* and *Ennead* unambiguously strengthen the *P*_A_ signal of *Algol*, because they have an impact of *z_x_* ≥ 1.0 and a significance of *Q*_z_ ≤ 0.2 with the ephemeris of Eq (11). The lucky prognoses of *Heliopolis* and *Enemy* are connected to *Algol* (*Q*_z_ ≤ 0.2), but they are not connected to the *P*_A_ signal (*z*_x_ < 1.0). In this section, we discuss these eight SWs in the order of their impact on the *P*_A_ signal, i.e. in the order of decreasing *z*_x_ with the ephemeris of Eq (11).

#### Horus

This SW has the largest impact *z*_x_ = +3.5 on the *P*_A_ signal and the highest significance of the above eight SWs (*Q*_z_ = 0.03, *n*_G_ = 14). The unit vectors **g**_i_ and **s**_i_ of lucky and unlucky prognoses with the ephemeris of Eq (11) are shown in Fig 1ab. Point Aa is at *ϕ* = 0 ≡ 0°. Points Ab, Ac and Ad are separated by Δ*ϕ* = 0.25 ≡ 90°. Only the **g**_i_ pointing between Ad ≡ −90° and Ab ≡ +90° strengthen the *P*_A_ signal. Twelve out of all fourteen **g**_i_ are within this interval (Fig 1a). The four Θ_i_ closest to Θ_R_ = 11° reach a high significance of *Q*_B_ = 0.006 (*n*_1_ = 4, *n*_2_ = 10, *N*_G_ = 177). The **g**_i_ pointing closest to Aa and giving the strongest impact on the *P*_A_ signal has the CC text [13]

*g*_i_(14, 2) ≡ +6°: *“It is the day of receiving the white crown by the Majesty of Horus; his Ennead is in great festivity.”*

The texts [12, 13] for the next best **g**_i_ closest to Aa are

*g*_i_(19, 12) ≡ +13°: *“Horus has returned complete, nothing is missing in it.”*

*g*_i_(27, 1) ≡ +19°: *“Peace on the part of Horus with Seth.”*

*g*_i_(24, 3) ≡ +19°: *“He has given his throne to his son, Horus, in front of Re.”*

*g*_i_(1, 7) ≡ +32°: *“Feast of entering into heaven and the two banks. Horus is jubilating.”*

*g*_i_(15, 11) ≡ +38°: *“Horus hears your words in the presence of every god and goddess on this day.”*

*g*_i_(27, 3) ≡ +38°: *“Judging Horus and Seth; stopping the fighting.”*

*g*_i_(18, 1) ≡ −38°: *“It is the day of magnifying the majesty of Horus more than his brother, …”*

*g*_i_(1, 9) ≡ +51°: *“Feast of Horus son of Isis and … his followers … day”*

*g*_i_(23, 7) ≡ −69°: *“Feast of Horus … on this day of his years in his very beautiful images.”*

*g*_i_(29, 3) ≡ −69°: *“White crown to Horus, and the red one to Seth.”*

*g*_i_(7, 9) ≡ +88°: *“The crew follow Horus in the foreign land, examining its list … therein when he smote him who rebelled against his master.”*

*g*_i_(1, 10) ≡ −120°: *“Horus … Osiris … Chentechtai … land”*

*g*_i_(28, 3) ≡ +164°: *“The gods are in jubilation and in joy when the will is written (lit. made) for Horus, …”*

These passages of lucky prognoses are suggestive of *Algol* at its brightest. The *“white crown”*, *Horus* having *“returned complete”* and *“entering into heaven”* (i.e. into the sky) are not easy to explain as symbols for the eclipse. Among the *g*_i_ of all 28 SWs, the *g*_i_ of *Horus* are the “best hit” on Aa (*z*_x_ = +3.5). If these *g*_i_ represent *Algol* at its brightest, then Aa is in the middle of this brightest phase and the thick line centered at Ac in Fig 1a outlines *Algol*’s eclipse. In this case, the *g*_i_(7, 9) ≡ +88° text may refer to an imminent eclipse and *“the will is written”* in *g*_i_(28, 3) ≡ +164° to the moment when the beginning of the eclipse is just becoming observable with naked eye. These passages could certainly describe naked eye observations of the regular changes of *Algol*.

Three **s**_i_ of *Horus* in Fig 1b concentrate close to Ad and reach *Q*_B_ = 0.07 (*n*_1_ = 3, *n*_2_ = 25, *N*_S_ = 105). The fourth vector **s**_i_ points close to Aa. Their CC texts [13] are

*s*_i_(26, 1) ≡ −107°: *“… It is the day of Horus fighting with Seth. …”*

*s*_i_(11, 11) ≡ −107°: *“Introducing the great ones by Re to the booth to see what he had observed through the eye of Horus the elder. They were with heads bent down when they saw the eye of Horus being angry in front of Re.”*

*s*_i_(20, 9) ≡ −69°: *“Mat judges in front of these gods who became angry in the island of the sanctuary of Letopolis. The Majesty of Horus revised it.”*

*s*_i_(5, 8) ≡ 6°: *“The Majesty of Horus is well when the red one sees his form. As for anybody who approaches it, anger will start on it.”*

If the *g*_i_ that described feasts were connected to the brightest phase of *Algol*, these *s*_i_ describing anger would have occurred after *Algol*’s eclipse. *“Horus is well”* for the last *s*_i_(5, 8) would seem natural for a lucky prognosis of *Horus* (as it should be close to Aa) but it is deemed unlucky for some other reasons. This type of “conflict of interest” prognoses may explain, why there are significant concentrations of directions accompanied by a few irregular directions (e.g. Fig 7c).

The *g*_i_ and *s*_i_ of *Horus* have *Q*_z_ > 0.2 with the ephemeris of Eq (12), and are therefore not connected to the *Moon*, except for some *g*_i_ texts mentioning both *Horus* and *Seth*. We argue that, as Leitz [12] also did, Mc ≡ 180° in Fig 1c coincides with the New *Moon* (see paragraph *Seth*). All the aforementioned lucky prognoses mentioning both *Horus* and *Seth* are close to Md ≡ −90° in Fig 1c, i.e. *g*_i_(27, 1) ≡ −82°, *g*_i_(27, 3) ≡ −73° and *g*_i_(29, 3) ≡ −48° with the ephemeris of Eq (12). The texts of these three days may describe the “luminosity competitions” between *Horus* and *Seth* which come to an end when more than half of the lunar disk becomes illuminated immediately after Md. The legend of the Contendings of *Horus* and *Seth*[14] (hereafter LE1) has inspired these descriptions. The text *“White crown to Horus, and the red one to Seth”* in *g*_i_(29, 3) would describe the brightening of *Horus* with *Algol* (Fig 1a: Θ = −69°) and the brightening of *Seth* (Fig 1c: Θ = −48°) with the approaching Full *Moon* at Ma. The most simple explanation for the context of these texts is that the lucky prognoses of *Horus* are connected to *Algol* at its brightest.

#### Re

The lucky prognoses reach *Q*_z_ = 0.07 (*n*_G_ = 32) with the ephemeris of Eq (11) and give the second largest impact *z*_x_ = +2.5 on the *P*_A_ signal (Fig 2a). Absence of small *Q*_B_ values, i.e. **g**_i_ concentrations, may indicate that *Re* (the *Sun*) was casually following the undertakings of *Horus*. The **s**_i_ of *Re* reach *Q*_z_ = 0.2 (*n*_S_ = 26) with the ephemeris of Eq (12), and explicitly avoid Ma, the proposed Full *Moon* phase (Fig 2d).

#### Wedjat

The lucky prognoses show weak periodicity (*Q*_z_ = 0.1, *n*_G_ = 4) with the ephemeris of Eq (11). They give the third largest impact *z*_x_ = +2.0 on the *P*_A_ signal (Fig 3a). However, their impact on the *P*_M_ signal is even larger, *z*_x_ = +2.9 (Fig 3c). *Wedjat* may represent *Algol* observed at its brightest close to the Full *Moon*. The **g**_i_ and **s**_i_ distributions of *Horus* and *Wedjat* are similar (Figs 1ab and 3ab) with the ephemeris of Eq (11). *Wedjat* is the Eye of Horus in Ancient Egyptian mythology.

#### Followers

The lucky prognoses have an impact of *z*_x_ = +1.4 on the *P*_A_ signal (Fig 4a). This periodicity is weak (*Q*_z_ = 0.2, *n*_G_ = 15). Six *s*_i_ reach *Q*_z_ = 0.01 (Fig 4b). The five **s**_i_ closest to Θ_R_ reach a high significance of *Q*_B_ = 0.003 (*n*_1_ = 5, *n*_2_ = 18, *N*_S_ = 105) and may refer to an approaching eclipse of *Algol*. These *s*_i_ also show a weak connection to the *Moon* (Fig 4d). It is tempting to suggest that *Followers* would be *Pleiades* following very close behind *Algol* in the revolving sky, e.g. in *g*_i_(7, 9) ≡ 88° *“The crew follow Horus in the foreign land”* (Figs 1a and 4a).

#### Sakhmet

The *g*_i_ and *s*_i_ reach *Q*_z_ = 0.06 (*n*_G_ = 4) and 0.05 (*n*_S_ = 3) with the ephemeris of Eq (11). The impact of *g*_i_ on the *P*_A_ signal is *z*_x_ = +1.3 (Fig 5a). The three *s*_i_ at Ad, after the proposed eclipse at Ac, are strongly connected to *Algol*, because they reach the most extreme significance in this study, *Q*_B_ = 0.0004 (*n*_1_ = 3, *n*_2_ = 6, *N*_S_ = 105). The texts [13] are

*s*_i_(27, 8) ≡ −95°: *“Re sets because the Majesty of the goddess Sakhmet is angry in the land of Temhu.”*

*s*_i_(13, 6) ≡ −82°: *“It is the day of the proceeding of Sakhmet to Letopolis. Her great executioners passed by the offerings of Letopolis on this day.”*

*s*_i_(7, 10) ≡ −82°: *“It is the day of the executioners of Sakhmet.”*

These three unlucky prognoses (Fig 5b) are immediately followed by lucky ones (Fig 5a). The **g**_i_ and **s**_i_ distributions of *Sakhmet* (Fig 5ab) resemble those of *Horus* (Fig 1ab) with the ephemeris of Eq (11). The Eye of Horus (*Wedjat*) was transformed into the vengeful goddess *Sakhmet* in the legend [14] of the Destruction of Mankind (hereafter LE2). The **s**_i_ vectors of *Horus*, *Wedjat* and *Sakhmet* point close to Ad which is after *Algol*’s proposed eclipse at Ac (Figs 1b, 3b and 5b), and may refer to the abrupt pacification of enraged *Sakhmet* in LE2.

#### Ennead

The lucky prognoses show weak periodicity (Fig 6a: *Q*_z_ = 0.1, *n*_G_ = 18) and an impact of *z*_x_ = +1.1 on the *P*_A_ signal with the ephemeris of Eq (11), as well as some concentration (*Q*_B_ = 0.02, *n*_1_ = 12, *n*_2_ = 63, *N*_G_ = 177). Ennead was a group of nine deities in Ancient Egyptian mythology. We discussed earlier, why *Followers* may have represented *Pleiades*. *Ennead* may have been another name for *Pleiades*, having the modern name “Seven sisters”. However, the number of *Pleiades* members visible with naked eye depends on the observing conditions and the observer, the maximum number of such members being fourteen [15, 16]. The unlucky prognoses of *Followers* could be connected to *Pleiades* following the disappearing *Algol* before eclipse (Fig 4b), while the unlucky prognoses of *Ennead* could be connected to *Algol* reappearing in front *Pleiades* after eclipse (Fig 6b). Furthermore, the lucky prognosis distributions of *Followers* and *Ennead* are very similar (Figs 4a and 6a).

#### Heliopolis

The lucky prognoses show weak periodicity with *P*_A_, but their impact on this signal is insignificant, *z*_x_ = +0.2, with the ephemeris of Eq (11).

#### Enemy

These lucky prognoses weaken the *P*_A_ signal, because their impact is *z*_x_ = −1.0 with the ephemeris of Eq (11).

### The Moon in lucky prognoses

We discuss the remaining other 20 SWs in this section and in sections

Algol in unlucky prognoses
The Moon in unlucky prognoses
No Algol or the Moon in lucky or unlucky prognoses

These SWs are discussed only briefly, because they are not connected to the *P*_A_ signal.

The lucky prognoses of *Earth*, *Heaven*, *Busiris*, *Rebel*, *Thoth* and *Onnophris* are connected to the *P*_M_ signal, because they have *z*_x_ ≥ 1.0 and *Q*_z_ ≤ 0.2 with the ephemeris of Eq (12). The lucky prognoses of *Nut* are weakly connected to the *Moon*.

#### Earth

These lucky prognoses reach the highest impact parameter value of this study, *z*_x_ = +5.3, on the *P*_M_ signal. This periodicity also reaches the highest Rayleigh test significance of all, *Q*_z_ = 0.001 (*n*_G_ = 19). The good moments on *Earth* occurred before and during Ma, the proposed Full *Moon* phase (Fig 7c). The unlucky prognoses also show a weak connection to *Algol* (Fig 7b: *Q*_z_ = 0.06, *n*_S_ = 5) and an even weaker connection to the *Moon* (Fig 7d: *Q*_z_ = 0.2, *n*_S_ = 5).

#### Heaven

The second largest impact *z*_x_ = +3.4 on the *P*_M_ signal comes from these lucky prognoses. Again, the good moments coincide with Ma, the proposed Full *Moon* phase (Fig 8c). This is significant periodicity (*Q*_z_ = 0.03, *n*_G_ = 19) combined with a very significant concentration (*Q*_B_ = 0.002, *n*_1_ = 12, *n*_2_ = 45, *N*_G_ = 177). The unlucky prognoses also show a weak connection to the *Moon* (Fig 8d: *Q*_z_ = 0.06, *n*_S_ = 4).

#### Busiris

The third largest impact on the *P*_M_ signal, *z*_x_ = +3.0, comes from the lucky prognoses of *Busiris*. This periodicity reaches *Q*_z_ = 0.05 (*n*_G_ = 4) with the ephemeris of Eq (12). And again, the lucky prognoses are close to Ma, the proposed Full *Moon* phase (Fig 9c)

#### Rebel

The lucky prognoses show weak periodicity (*Q*_z_ = 0.2, *n*_G_ = 3) with the ephemeris of Eq (12) and have an impact of *z*_x_ = 1.6 on the *P*_M_ signal.

#### Thoth and Onnophris

The lucky prognoses of these SW have a weaker impact on the *P*_M_ signal, i.e. 1.0 ≤ *z*_x_ ≤ 1.3 with the ephemeris Eq (12).

#### Nut

The lucky prognoses show a weak connection to the *Moon*. They have no impact on *P*_M_, because *z*_x_ = −0.1 with the ephemeris of Eq (12).

### Algol in unlucky prognoses

The *P*_A_ and *P*_M_ signals were detected from the lucky prognoses *g*_i_[10, 11]. It is therefore self–evident that the unlucky prognoses *s*_i_ had no impact on these two signals. However, this does not rule out the possibility that the *s*_i_ of some SW may be connected to *Algol* or the *Moon*. Most of these **s**_i_ vectors point away from Aa or Ma, i.e. *z*_x_ < 0 with the ephemerides of Eqs (11) or (12). *Man* and *Flame* are the only exceptions to this general rule (*z*_x_ ≥ 0).

#### Heart

The unlucky prognoses have *z*_x_ = −3.1 with the ephemeris of Eq (11). They point towards Ac, the proposed eclipse phase of *Algol* (Fig 10b). This periodicity reaches a significance of *Q*_z_ = 0.04 (*n*_S_ = 5) and *Q*_B_ = 0.04 (*n*_1_ = 5, *n*_2_ = 39, *N*_S_ = 105).

#### Nun

The three unlucky prognoses of this SW reach *Q*_z_ = 0.06 and a high significance of *Q*_B_ = 0.003 (*n*_1_ = 3, *n*_2_ = 11, *N*_S_ = 105) with the ephemeris of Eq (11). They also show a weaker connection to the *Moon*.

### The Moon in unlucky prognoses

We will first discuss the unlucky prognoses of SWs having negative *z*_x_ values with the ephemeris of Eq (12), and then the two exceptions of *Man* and *Flame*.

#### Seth

“See you on the dark side of the Moon” sums up the unlucky prognoses of *Seth* (Fig 11d). The significance is *Q*_z_ = 0.05 (*n*_S_ = 9) with the ephemeris of Eq (12). Leitz [12] has argued that the following texts [13] at two consecutive days

*s*_i_(16, 7) ≡ 173°: *“Do not look, darkness being on this day (or, do not see darkness on this day).”*

*s*_i_(17, 7) ≡ 185°: *“Do not pronounce the name of Seth on this day.”*

take place during the New *Moon*. The **s**_i_ vectors of these two particular texts point at the opposite sides of Mc ≡ 180°, which supports both our “prediction” formula of Eq (12) and Leitz’ attribution [12] of the texts to the New *Moon*. We conclude that *Seth* is connected to the *Moon* and strongly suggest that Mc computed with Eq (12) is close to the New *Moon*. Hence, the Full *Moon* is close to Ma.

#### Osiris

The four unlucky prognoses of this SW also point to the dark side of the *Moon*, assuming that Mc is close to the New *Moon* (Fig 12d). The significance estimates are *Q*_z_ = 0.05 (*n*_S_ = 4) and *Q*_B_ = 0.02 (*n*_1_ = 3, *n*_2_ = 15, *N*_S_ = 105) with the ephemeris of Eq (12).

#### Abydos and Lion

These unlucky prognoses show a weak connection to the *Moon*.

#### Man

The significance estimates for the unlucky prognoses are *Q*_z_ = 0.02 (*n*_S_ = 6) and *Q*_B_ = 0.009 (*n*_1_ = 5, *n*_2_ = 23, *N*_S_ = 105) with the ephemeris Eq (12). These unlucky moments of *Man* concentrate on a few days after Ma, the proposed Full *Moon* phase (Fig 13d).

#### Flame

The significance estimates for these unlucky prognoses are *Q*_z_ = 0.03 (*n*_S_ = 4) and *Q*_B_ = 0.003 (*n*_1_ = 4, *n*_2_ = 17, *N*_S_ = 105) with the ephemeris of Eq (12).

### No Algol or the Moon in lucky or unlucky prognoses

#### Eye, Fire, Majesty, Shu and Sobek

These SWs are not connected to *Algol* or the *Moon*, because their *g*_i_ and *s*_i_ have *Q*_z_ > 0.2 with the ephemerides of Eqs (11) and (12).

### Some general remarks

This concludes our analysis of 28 SWs. Numerous other [7] SWs in CC need to be analysed in the future. Combining the inverse relations of Eqs (3) and (4) to the ephemerides of Eqs (11) and (12) will have countless applications. For example, the first eclipse of *Algol* would have occurred on *t*(2.6, 1) = 1.96 at *D* = 2.1 in *M* = 1 or the last New *Moon* on *t*(14.6, 12) = 343.9 at *D* = 14.6 in *M* = 12. Any question about CC can now be studied within this precise framework, e.g. was some meaning given to the nights when an eclipse of *Algol* (Eq (11): *ϕ* = 0.5) coincided with the New *Moon* (Eq (12): *ϕ* = 0.5)?

## Discussion

Previously, we [11] applied four tests to the astrophysical hypothesis

*H*_1_: *“Period P_A_ = 2.^d^850 in CC was P_orb_ of Algol.”*

This is a summary of those tests:

test i: The mass transfer in this binary system should have increased the period in the past three millennia. The period value in CC is the first evidence for such an increase since Goodricke [17] discovered this periodicity over two centuries ago.
test ii: The period change of 0.017 days from 2.850 to 2.867 days gives a reasonable estimate for the rate of this mass transfer.
test iii: If eclipses were observed in Ancient Egypt, the orbital plane of the Algol A–B system must be nearly perpendicular to that of the Algol AB–C system [18, 19].
test iv: *Algol* and the *Moon* are the most probable objects, where naked eye observers could have discovered periodicity that we could then rediscover in CC.

tests i and iv supported *H*_1_, while tests ii and iii indicated that it could be true.

**Fig 14:**
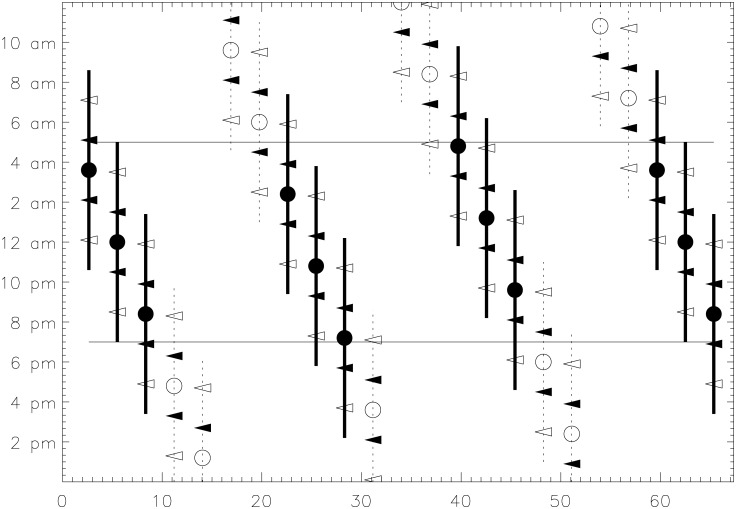
Eclipses of Algol with *P*_A_ = 2.85 days. The horizontal continuous lines show the beginnings and ends of 10 hours long nights. The filled and open circles denote mid eclipse epochs occurring inside and outside such nights. The *T*_A1_ = 10 hour time intervals of eclipses are denoted with thick continuous or thin dashed lines. The tilted open and closed triangles show the *T*_A2_ = 7 and *T*_A3_ = 3 hour limits.

*Algol*’s observable night time mid eclipse epochs occur in groups of three separated with a period of 19 days and we also discovered this period in CC [11]. This phenomenon is displayed in Fig 14. First, a mid eclipse epoch occurs in the end of the night. After three days, the next one occurs close to midnight. After another three days, a mid eclipse epoch occurs in the beginning of the night. Then, the next observable night-time mid eclipse epoch occurs after 13 days. Naked eye observations could easily lead to the discovery of this 3 + 3 + 13 days regularity. One could speculate that this is one of the reasons, why the prime number 13 is still considered unlucky. This would be consistent with our result that, if the brightest phases of *Algol* were considered lucky then the eclipses (i.e. the dimmer phases) were considered unlucky. The 2.85 days period is exactly equal to 57/20 days. This means that after 57 = 3 × 19 days the eclipses returned exactly to the same moment of the night (see Fig 14). All *D* = 1 days in CC have a prognosis combination “GGG”, while all *D* = 20 days have “SSS”. Perhaps this regular separation of 19 days was also inspired by *Algol*.

Only a skilled naked eye observer would have been able to discover the minor exceptions from the 3 + 3 + 13 days regularity. *Algol*’s eclipses last *T*_A1_ = 10 hours. Naked eye can detect brightness differences of 0.^m^1 in ideal observing conditions. Hence, an eclipse detection is theoretically *possible* for *T*_A2_ = 7 hours when *Algol* is more than 0.^m^1 dimmer than its brightest suitable comparison star *γ* And (Fig 14: tilted open triangle limits). This detection could become *certain* for *T*_A3_ = 3 hours when *Algol* is also at least 0.^m^1 dimmer than all its other suitable comparison stars *ζ* Per, *ϵ* Per, *γ* Per, *δ* Per and *β* Tri (Fig 14: tilted closed triangle limits). During the 57 days eclipse repetition cycle, only two mid eclipse epochs outside the 10 hour night time limits would qualify as *certain* observable eclipses (Fig 14: open circles at 19th and 48th days). However, a *certain* detection of these two events would have been very difficult so close to dawn and dusk. The same argument is true for three additional *possible* eclipse detections (Fig 14: open circles at 11th, 31st and 54th days).

Here, our statistical analysis of SWs giving the largest impact on the *P*_A_ signal reveals that *Algol* was represented as *Horus*. The lucky prognoses were most likely connected to *Algol*’s brightest phase. *Sakhmet* may have represented *Algol* after eclipses, and *Wedjat* during periods close to the Full *Moon*. To the Ancient Egyptians, *Algol*’s cycle may have symbolised the familiar events of LE1 and LE2. At Aa, *Re* sends the Eye of Horus (*Wedjat*) to destroy the rebels, as in LE2. At Ab, *Horus* enters the *“foreign land”* in *g*_i_(7, 9), where he *“smote him who rebelled”*, as in LE1 or LE2. The *“will is written”* for him in *g*_i_(28, 3) at the beginning of an eclipse—the only **g**_i_ vector of *Horus* overlapping the thick line centered at Ac in Fig 1a. After an eclipse, *Wedjat* returns as *Sakhmet* who is pacified immediately after Ad, as in LE2. And a new cycle begins.

*Followers* and *Ennead* may have represented *Pleiades*. Thus, these two, together with *Horus*, *Re*, *Wedjat* and *Sakhmet*, give the largest impact on the *P*_A_ signal.

The two periods, *P*_A_ and *P*_M_, regulate the assignment of mythological texts to specific days of the year. The *Moon* strongly regulates the times described as lucky for *Heaven* and for *Earth* (Figs 7c and 8c). The unlucky prognoses of *Seth* are clearly associated with the phases of the *Moon* (Fig 11d). Other SWs follow *P*_A_ or *P*_M_, like *Busiris*, *Heart*, *Osiris* and *Man* (Figs 9, 10, 12 and 13). We show no figures for *Heliopolis*, *Enemy*, *Rebel*, *Thoth*, *Onnophris*, *Nut*, *Nun*, *Abydos*, *Lion* and *Flame* which also reach *Q*_z_ ≤ 0.2 with *P*_A_ or *P*_M_. All these regularities can not simply be dismissed as a coincidence, let alone with the possible errors of *σ*_*t*_ ≈ ±0.5 or ±1.5 days.

## Conclusions

What was the origin of the phenomenon that occurred every third day, but always 3 hours and 36 minutes earlier than before, and caught the attention of Ancient Egyptians? Our statistical analysis leads us to argue that the mythological texts of CC contain astrophysical information about *Algol*. In 1596, Fabricius discovered the first variable star, *Mira*. Holwarda determined its eleven month period 44 years later. In 1669, Montanari discovered the second variable star, *Algol*. Goodricke [17] determined the 2.867 days period of *Algol* in 1783. All these astronomical discoveries were made with naked eye. Since then, they have become milestones of natural sciences. Our statistical analysis of CC confirms that all these milestones should be shifted about three millennia backwards in time.

## Supporting Information

S1 Fig Text of Cairo Calendar page rto VIII. Inside our superimposed rectangle is the hieratic writing for the word *Horus*. Reprinted from Leitz [12] under a CC BY license, with permission from Harrassowitz Verlag, original copyright [1994].
(EPS)

S1 Table Analysis results for all SWs. Day (*D*), month (*M*) of lucky (*g_i_*) and unlucky (*s_i_*) time points, their phase (ϕ*_i_*), phase angle (Θ_i_), direction of their **R** vector (Θ_R_) and differences ΔΘ_i_ = Δ_i_ − Θ_R_ with Eq (11) for *P_A_* = 2.85 days and Eq (12) for PM = 29.6 days. The binomial distribution parameters are *n*_1_, *n*_2_, *q_B_* for *Q*_B_. Note that the parameters are given in the order of increasing ΔΘ_i_, *n*_1_ and *n*_2_. All values mentioned in text are marked in bold. We also make available the code of a Python 3.0 program *tableS1.py* which can be downloaded on Dryad (http://dx.doi.org/10.5061/dryad.tj4qg). This program can be used to reproduce and replicate all analysis results given in S1 Table.
(PDF)

## References

[1] Leitz C. Studien zur Ägyptischen Astronomie. Wiesbaden, Germany: Harrassowitz; 1989.

[2] Wells RA. “Re and the Calendars”, in Revolution in Time: Studies in Ancient Egyptian Calendrics. San Antonio, USA: Van Siclen Books; 1994.

[3] Clagett M. Ancient Egyptian Science, Vol 2: Calendars, Clocks and Astronomy. Philadephia, USA: American Philosophical Society; 1995.

[4] Smith DG. Solar Eclipse Events in the New Kingdom. Part 2—Astronomical Analysis. Egyptological Journal, Articles. 2012 10;6.

[5] Wells RA. Horoscopes, in The Oxford Encyclopedia of Ancient Egypt, Vol 2 Oxford, England: Oxford University Press; 2001.

[6] Krauss R. The Eye of Horus and the Planet Venus: Astronomical and Mythological References. Altes Orient und Altes Testament. 2002;297.

[7] Hardy PA. The Cairo Calendar as a Stellar Almanac. Archaeoastronomy. 2002 6;17:48–63.

[8] Brunner-Traut E. Mythos im Alltag. Antaios. 1970;12:332–347.

[9] Troy L. Have A Nice Day. Boreas, Uppsala Studies in Ancient Mediterranean and near Eastern Civilizations. 1989;20:127–147.

[10] Porceddu S, Jetsu L, Markkanen T, Toivari-Viitala J. Evidence of Periodicity in Ancient Egyptian Calendars of Lucky and Unlucky Days. Cambridge Archaeological Journal. 2008;18:3:327–339. 10.1017/S0959774308000395

[11] Jetsu L, Porceddu S, Lyytinen J, Kajatkari P, Lehtinen J, Markkanen T, et al Did the Ancient Egyptians Record the Period of the Eclipsing Binary Algol—The Raging One? ApJ. 2013 8;773:1 10.1088/0004-637X/773/1/1

[12] Leitz C. Tagewählerei Das Buch und verwandte Texte. Wiesbaden, Germany: Harrassowitz; 1994.

[13] Bakir A. The Cairo Calendar No. 86637. Cairo, Egypt: Government Printing Office; 1966.

[14] Lichtheim M. Ancient Egyptian Literature—Volume II: The New Kingdom. University of California Press, USA; 1976.

[15] Winnecke A. on the visibility of stars in the Pleiades to the naked eye. MNRAS. 1878 12;39:146 10.1093/mnras/39.2.146

[16] Adams WB. The Hands of the Pleiades: The Celestial Clock in the Classical Arabic Poetry of Dhū al-Rumma. In: Corsini EM, editor. The Inspiration of Astronomical Phenomena VI. vol. 441 of Astronomical Society of the Pacific Conference Series; 2011. p. 311.

[17] Goodricke J. a Series of Observations on, and a Discovery of, the Period of the Variation of the Light of the Bright Star in the Head of Medusa, Called Algol. in a Letter from John Goodricke, Esq. to the Rev. Anthony Shepherd, D. D. F. R. S. and Plumian Professor at Cambridge. Royal Society of London Philosophical Transactions Series I. 1783;73:474–482.

[18] Zavala RT, Hummel CA, Boboltz DA, Ojha R, Shaffer DB, Tycner C, et al The Algol Triple System Spatially Resolved at Optical Wavelengths. ApJ. 2010 5;715:L44–L48. 10.1088/2041-8205/715/1/L44

[19] Baron F, Monnier JD, Pedretti E, Zhao M, Schaefer G, Parks R, et al Imaging the Algol Triple System in the H Band with the CHARA Interferometer. ApJ. 2012 6;752:20 10.1088/0004-637X/752/1/20

