# Correction: Shifting Milestones of Natural Sciences: The Ancient Egyptian Discovery of Algol's Period Confirmed

**DOI:** 10.1371/journal.pone.0149042

**Published:** 2016-02-04

**Authors:** The PLOS ONE Staff

## Notice of Republication

This article was republished on January 25, 2016, to correct capitalization errors in notations throughout the paper that were introduced during the typesetting process. In addition, a sentence was corrected in the Discussion section, and Table 6 was replaced with S1 Table. The publisher apologizes for the errors. Please download this article again to view the correct version. The originally published, uncorrected article and the republished, corrected article are provided here for reference.

## Supporting Information

S1 File Originally published, uncorrected article. (PDF)

S2 File Republished, corrected article. (PDF)
