# Supplementary material for: Shifting Milestones of Natural Sciences: The Ancient Egyptian Discovery of Algol’s Period Confirmed
Source: PLoS One. 2015 Dec 17;10(12):e0144140. doi: 10.1371/journal.pone.0144140 (PMC4683080; doi:10.1371/journal.pone.0144140)
Supplement: S1 Table — Day (D), month (M) of lucky (gi) and unlucky (si) time points, their phase (ϕi), phase angle (Θi), direction of their R vector (ΘR) and differences ΔΘi = Δi − ΘR with Eq (11) for PA = 2.85 days and Eq (12) for PM = 29.6 days. The binomial distribution parameters are n 1, n 2, qB for Q B. Note that the parameters are given in the order of increasing ΔΘi, n 1 and n 2. All values mentioned in text are marked in bold. We also make available the code of a Python 3.0 program tableS1.py which can be downloaded on Dryad (http://dx.doi.org/10.5061/dryad.tj4qg). This program can be used to reproduce and replicate all analysis results given in S1 Table. (PDF) [file pone.0144140.s002.pdf]

Horus: $n_G = \mathbf{14}$, $N_G = \mathbf{177}$, $q_B = 0.0791$

$P = 2.85$, $\Theta_R = \mathbf{11}$<br>Periodicity: $z = 3.6$, $Q_z = \mathbf{0.03}$, $z_x = \mathbf{3.5}$								
D	M	g_i	ϕ_i	Θ_i	$\Delta\Theta_i$	n_1	n_2	Q_B
19	12	348.33	0.04	**13**	1	1	4	0.2808
14	2	43.33	0.02	**6**	5	2	5	0.0532
27	1	26.33	0.05	**19**	7	3	9	0.0289
24	3	83.33	0.05	**19**	7	**4**	**10**	**0.0056**
1	7	180.33	0.09	**32**	20	5	29	0.0747
27	3	86.33	0.11	**38**	26	6	40	0.0928
15	11	314.33	0.11	**38**	26	7	43	0.0503
1	9	240.33	0.14	**51**	39	8	57	0.0788
18	1	17.33	-0.11	**-38**	49	9	68	0.0869
7	9	246.33	0.25	**88**	77	10	95	0.2180
29	3	88.33	-0.19	**-69**	81	11	98	0.1515
23	7	202.33	-0.19	**-69**	81	12	99	0.0912
1	10	270.33	-0.33	**-120**	131	13	142	0.3332
28	3	87.33	0.46	**164**	153	14	155	0.3428

$P = 29.60$, $\Theta_R = -75$<br>No periodicity								
D	M	g_i	ϕ_i	Θ_i	$\Delta\Theta_i$	n_1	n_2	Q_B
27	3	86.33	-0.20	**-73**	2	1	2	0.1519
27	1	26.33	-0.23	**-82**	8	2	7	0.1006
28	3	87.33	-0.17	-60	14	3	16	0.1279
29	3	88.33	-0.13	**-48**	26	4	25	0.1311
23	7	202.33	-0.28	-102	27	5	26	0.0503
24	3	83.33	-0.30	-109	34	6	36	0.0613
19	12	348.33	-0.35	-126	51	7	54	0.1322
1	7	180.33	-0.03	-9	65	8	67	0.1581
1	9	240.33	0.00	0	75	9	79	0.1712
1	10	270.33	0.01	5	80	10	85	0.1337
15	11	314.33	-0.50	-180	105	11	110	0.2529
18	1	17.33	0.47	168	117	12	119	0.2317
7	9	246.33	0.20	73	148	13	148	0.3890
14	2	43.33	0.35	124	161	14	159	0.3785

Horus: $n_S = 4$, $N_S = \mathbf{105}$, $q_B = 0.0381$

$P = 2.85$, $\Theta_R = -75$<br>Weak periodicity: $z = 2.0$, $Q_z = 0.1$, $z_x = 0.1$								
D	M	s_i	ϕ_i	Θ_i	$\Delta\Theta_i$	n_1	n_2	Q_B
20	9	259.33	-0.19	**-69**	5	1	5	0.1765
26	1	25.33	-0.30	**-107**	33	2	21	0.1897
11	11	310.33	-0.30	**-107**	33	**3**	**25**	**0.0681**
5	8	214.33	0.02	**6**	81	4	49	0.1159

$P = 29.60$, $\Theta_R = -153$<br>No periodicity								
D	M	s_i	ϕ_i	Θ_i	$\Delta\Theta_i$	n_1	n_2	Q_B
20	9	259.33	-0.36	-129	24	1	23	0.5907
26	1	25.33	-0.26	-95	58	2	42	0.4788
11	11	310.33	0.37	132	76	3	57	0.3705
5	8	214.33	0.12	44	163	4	97	0.5077

Re: $n_G = \mathbf{32}$, $N_G = 177$, $q_B = 0.1808$

$P = 2.85$, $\Theta_R = 17$ Weak periodicity: $z = 2.7$, $Q_z = \mathbf{0.07}$, $z_x = \mathbf{2.5}$								
D	M	g_i	ϕ_i	Θ_i	$\Delta\Theta_i$	n_1	n_2	Q_B
24	3	83.33	0.05	19	2	1	2	0.3289
1	5	120.33	0.04	13	5	2	6	0.2975
17	2	46.33	0.07	25	8	3	9	0.2124
25	10	294.33	0.09	32	14	4	24	0.6537
21	3	80.33	0.00	0	17	5	27	0.5544
9	11	308.33	0.00	0	17	6	29	0.4305
27	3	86.33	0.11	38	21	7	31	0.3233
24	5	143.33	0.11	38	21	8	32	0.2098
8	10	277.33	0.12	44	27	9	38	0.2386
10	3	69.33	0.14	51	33	10	46	0.3137
25	12	354.33	0.14	51	33	11	49	0.2631
21	1	20.33	-0.05	-19	36	12	50	0.1807
3	2	32.33	0.16	57	40	13	54	0.1655
19	8	228.33	-0.07	-25	42	14	60	0.1845
2	6	151.33	-0.09	-32	49	15	67	0.2200
6	2	35.33	0.21	76	59	16	76	0.2921
10	12	339.33	-0.12	-44	61	17	82	0.3072
19	1	18.33	0.25	88	71	18	87	0.3031
9	2	38.33	0.26	95	78	19	96	0.3711
2	1	1.33	0.28	101	84	20	100	0.3472
29	3	88.33	-0.19	-69	87	21	106	0.3591
24	12	353.33	-0.21	-76	93	22	112	0.3704
9	4	98.33	0.32	114	97	23	113	0.2997
16	4	105.33	-0.23	-82	99	24	116	0.2657
9	1	8.33	-0.26	-95	112	25	122	0.2771
3	5	122.33	-0.26	-95	112	26	124	0.2323
3	10	272.33	0.37	133	115	27	128	0.2173
13	4	102.33	-0.28	-101	118	28	131	0.1914
25	5	144.33	0.46	164	147	29	152	0.4066
10	2	39.33	-0.39	-139	156	30	156	0.3859
1	4	90.33	-0.49	-177	166	31	164	0.4233
1	6	150.33	-0.44	-158	175	32	172	0.4598

$P = 29.60$, $\Theta_R = -4$ No periodicity								
D	M	g_i	ϕ_i	Θ_i	$\Delta\Theta_i$	n_1	n_2	Q_B
2	6	151.33	-0.01	-2	2	1	4	0.5496
3	2	32.33	-0.03	-9	5	2	9	0.5038
3	5	122.33	0.01	5	9	3	16	0.5731
1	6	150.33	-0.04	-14	10	4	18	0.4148
1	5	120.33	-0.05	-19	15	5	26	0.5185
1	4	90.33	-0.07	-24	20	6	31	0.4991
2	1	1.33	-0.07	-26	22	7	34	0.4192
6	2	35.33	0.08	27	31	8	45	0.5816
3	10	272.33	0.08	30	34	9	49	0.5372
29	3	88.33	-0.13	-48	44	10	62	0.7050
25	12	354.33	-0.15	-53	49	11	67	0.6865
25	10	294.33	-0.17	-63	59	12	75	0.7248
24	12	353.33	-0.18	-65	61	13	77	0.6531
9	1	8.33	0.16	59	63	14	78	0.5581
9	2	38.33	0.18	64	68	15	85	0.5853
27	3	86.33	-0.20	-73	68	16	87	0.5135
9	4	98.33	0.20	73	77	17	94	0.5414
10	2	39.33	0.21	76	80	18	98	0.5115
25	5	144.33	-0.24	-87	83	19	102	0.4829
10	3	69.33	0.22	81	85	20	104	0.4192
8	10	277.33	0.25	90	94	21	113	0.4827
24	5	143.33	-0.28	-99	95	22	114	0.4047
24	3	83.33	-0.30	-109	105	23	124	0.4824
9	11	308.33	0.30	107	112	24	128	0.4575
13	4	102.33	0.34	122	126	25	139	0.5460
10	12	339.33	0.35	124	129	26	142	0.5057
21	3	80.33	-0.40	-146	141	27	148	0.5128
19	8	228.33	-0.40	-146	141	28	149	0.4435
21	1	20.33	-0.43	-155	151	29	153	0.4216
16	4	105.33	0.44	158	163	30	162	0.4741
17	2	46.33	0.45	161	165	31	163	0.4089
19	1	18.33	-0.50	-180	176	32	173	0.4740

Re: $n_S = \mathbf{26}$, $N_S = 105$, $q_B = 0.2476$

$P = 2.85$, $\Theta_R = -124$ No periodicity								
D	M	s_i	ϕ_i	Θ_i	$\Delta\Theta_i$	n_1	n_2	Q_B
23	1	22.33	-0.35	-126	3	1	1	0.2476
20	3	79.33	-0.35	-126	3	2	2	0.0613
15	12	344.33	-0.37	-133	9	3	8	0.3155
20	5	139.33	-0.30	-107	16	4	13	0.4078
11	11	310.33	-0.30	-107	16	5	15	0.3057
10	6	159.33	-0.28	-101	23	6	19	0.3233
27	2	56.33	-0.42	-152	28	7	21	0.2480
27	8	236.33	-0.26	-95	29	8	24	0.2253
7	2	36.33	-0.44	-158	34	9	25	0.1428
7	10	276.33	-0.23	-82	41	10	33	0.2880
24	2	53.33	-0.47	-171	47	11	34	0.2011
12	1	11.33	-0.21	-76	48	12	36	0.1586
9	3	68.33	-0.21	-76	48	13	37	0.1045
26	5	145.33	-0.19	-69	54	14	40	0.0969
19	4	108.33	-0.18	-63	60	15	42	0.0751
16	11	315.33	0.46	164	72	16	49	0.1336
3	12	332.33	0.42	152	85	17	58	0.2531
22	1	21.33	0.30	107	129	18	78	0.6766
22	10	291.33	0.04	13	136	19	84	0.7145
6	4	95.33	0.26	95	142	20	85	0.6444
11	12	340.33	0.23	82	154	21	90	0.6623
7	7	186.33	0.19	69	167	22	96	0.6993
20	2	49.33	0.12	44	168	23	98	0.6542
11	10	280.33	0.18	63	173	24	101	0.6295
21	9	260.33	0.16	57	180	25	104	0.6051
18	11	317.33	0.16	57	180	26	105	0.5375

$P = 29.60$, $\Theta_R = -177$ Weak periodicity: $z = 1.7$, $Q_z = \mathbf{0.2}$, $z_x = -1.7$								
D	M	s_i	ϕ_i	Θ_i	$\Delta\Theta_i$	n_1	n_2	Q_B
15	12	344.33	-0.49	-175	2	1	3	0.5741
16	11	315.33	-0.47	-167	10	2	13	0.8693
19	4	108.33	-0.46	-165	12	3	14	0.7120
20	2	49.33	-0.45	-163	15	4	16	0.5864
20	3	79.33	-0.44	-158	20	5	19	0.5249
20	5	139.33	-0.41	-148	29	6	23	0.5209
22	1	21.33	-0.40	-143	34	7	25	0.4279
18	11	317.33	-0.40	-143	34	8	27	0.3464
11	12	340.33	0.38	137	46	9	34	0.4737
23	1	22.33	-0.36	-131	46	10	35	0.3612
11	11	310.33	0.37	132	51	11	39	0.3668
11	10	280.33	0.35	127	56	12	43	0.3715
21	9	260.33	-0.32	-116	61	13	46	0.3434
24	2	53.33	-0.32	-114	63	14	48	0.2880
22	10	291.33	-0.28	-99	78	15	55	0.3824
12	1	11.33	0.26	95	88	16	62	0.4728
10	6	159.33	0.26	95	88	17	63	0.3877
27	2	56.33	-0.22	-77	100	18	72	0.5265
26	5	145.33	-0.21	-75	102	19	74	0.4720
7	10	276.33	0.22	78	105	20	75	0.3938
9	3	68.33	0.19	68	114	21	79	0.3955
7	7	186.33	0.18	64	119	22	80	0.3245
27	8	236.33	-0.13	-48	129	23	87	0.3985
7	2	36.33	0.11	39	143	24	93	0.4470
3	12	332.33	0.11	39	143	25	94	0.3780
6	4	95.33	0.10	37	146	26	97	0.3574

Wedjat: $n_G = \mathbf{4}$, $N_G = 177$, $q_B = 0.0226$

$P = 2.85$, $\Theta_R = -8$ Weak periodicity: $z = 2.1$, $Q_z = \mathbf{0.1}$, $z_x = \mathbf{2.0}$								
D	M	g_i	ϕ_i	Θ_i	$\Delta\Theta_i$	n_1	n_2	Q_B
1	5	120.33	0.04	13	21	1	29	0.4846
6	9	245.33	-0.11	-38	30	2	43	0.2537
30	10	299.33	-0.16	-57	49	3	73	0.2285
3	2	32.33	0.16	57	65	4	84	0.1228

$P = 29.60$, $\Theta_R = 6$ Periodicity: $z = 2.9$, $Q_z = 0.05$, $z_x = \mathbf{2.9}$								
D	M	g_i	ϕ_i	Θ_i	$\Delta\Theta_i$	n_1	n_2	Q_B
30	10	299.33	-0.01	-2	8	1	12	0.2399
3	2	32.33	-0.03	-9	15	2	20	0.0742
1	5	120.33	-0.05	-19	25	3	36	0.0474
6	9	245.33	0.17	61	55	4	72	0.0804

Wedjat: $n_S = 1$, $N_S = 105$, $q_B = 0.0095$

$P = 2.85$, $\Theta_R = -101$ No periodicity								
D	M	s_i	ϕ_i	Θ_i				
10	6	159.33	-0.28	-101				

$P = 29.60$, $\Theta_R = 95$ No periodicity								
D	M	s_i	ϕ_i	Θ_i				
10	6	159.33	0.26	95				

Followers: $n_G = \mathbf{15}$, $N_G = 177$, $q_B = 0.0847$

$P = 2.85$, $\Theta_R = 22$ Weak periodicity: $z = 1.6$, $Q_z = \mathbf{0.2}$, $z_x = \mathbf{1.4}$								
D	M	g_i	ϕ_i	Θ_i	$\Delta\Theta_i$	n_1	n_2	Q_B
21	5	140.33	0.05	19	3	1	3	0.2333
1	5	120.33	0.04	13	9	2	12	0.2705
29	11	328.33	0.02	6	15	3	25	0.3567
8	10	277.33	0.12	44	23	4	35	0.3447
1	9	240.33	0.14	51	29	5	41	0.2651
3	2	32.33	0.16	57	35	6	50	0.2472
21	1	20.33	-0.05	-19	40	7	53	0.1591
6	9	245.33	-0.11	-38	59	8	77	0.3277
16	8	225.33	-0.12	-44	66	9	81	0.2468
13	10	282.33	-0.12	-44	66	10	82	0.1551
19	1	18.33	0.25	88	67	11	84	0.0976
3	10	272.33	0.37	133	111	12	123	0.3491
9	10	278.33	0.47	171	149	13	154	0.5476
10	2	39.33	-0.39	-139	160	14	160	0.4909
1	4	90.33	-0.49	-177	162	15	163	0.4088

$P = 29.60$, $\Theta_R = 57$ No periodicity								
D	M	g_i	ϕ_i	Θ_i	$\Delta\Theta_i$	n_1	n_2	Q_B
6	9	245.33	0.17	61	4	1	5	0.3577
10	2	39.33	0.21	76	18	2	20	0.5148
3	10	272.33	0.08	30	28	3	30	0.4731
8	10	277.33	0.25	90	33	4	38	0.4041
9	10	278.33	0.28	103	45	5	48	0.3849
1	9	240.33	0.00	0	57	6	62	0.4305
3	2	32.33	-0.03	-9	67	7	73	0.4249
29	11	328.33	-0.03	-9	67	8	75	0.3020
1	5	120.33	-0.05	-19	76	9	86	0.3047
1	4	90.33	-0.07	-24	81	10	91	0.2413
13	10	282.33	0.42	151	94	11	106	0.2865
16	8	225.33	0.49	178	121	12	131	0.4333
19	1	18.33	-0.50	-180	123	13	134	0.3475
21	1	20.33	-0.43	-155	147	14	156	0.4522
21	5	140.33	-0.38	-136	167	15	171	0.4838

Followers: $n_S = \mathbf{6}$, $N_S = \mathbf{105}$, $q_B = 0.0571$

$P = 2.85$, $\Theta_R = 82$ Periodicity: $z = 4.3$, $Q_z = \mathbf{0.01}$, $z_x = 0.1$								
D	M	s_i	ϕ_i	Θ_i	$\Delta\Theta_i$	n_1	n_2	Q_B
17	8	226.33	0.23	82	0	1	3	0.1618
11	12	340.33	0.23	82	0	2	4	0.0181
7	7	186.33	0.19	69	13	3	11	0.0218
11	10	280.33	0.18	63	19	4	15	0.0087
21	9	260.33	0.16	57	25	**5**	**18**	**0.0028**
11	1	10.33	0.44	158	76	6	42	0.0311

$P = 29.60$, $\Theta_R = 134$ Weak periodicity: $z = 1.7$, $Q_z = 0.2$, $z_x = -0.8$								
D	M	s_i	ϕ_i	Θ_i	$\Delta\Theta_i$	n_1	n_2	Q_B
11	12	340.33	0.38	137	3	1	3	0.1618
11	10	280.33	0.35	127	7	2	7	0.0566
11	1	10.33	0.23	83	50	3	31	0.2598
17	8	226.33	-0.47	-170	57	4	37	0.1590
7	7	186.33	0.18	64	70	5	48	0.1378
21	9	260.33	-0.32	-116	110	6	74	0.2477

Sakhmet: $n_G = \mathbf{4}$, $N_G = 177$, $q_B = 0.0226$

$P = 2.85$, $\Theta_R = -46$ Weak periodicity: $z = 2.7$, $Q_z = \mathbf{0.06}$, $z_x = \mathbf{1.3}$								
D	M	g_i	ϕ_i	Θ_i	$\Delta\Theta_i$	n_1	n_2	Q_B
29	5	148.33	-0.14	-51	4	1	6	0.1282
9	5	128.33	-0.16	-57	11	2	12	0.0290
16	4	105.33	-0.23	-82	36	3	45	0.0813
1	5	120.33	0.04	13	59	4	70	0.0741

$P = 29.60$, $\Theta_R = 22$ No periodicity								
D	M	g_i	ϕ_i	Θ_i	$\Delta\Theta_i$	n_1	n_2	Q_B
1	5	120.33	-0.05	-19	41	1	51	0.6883
9	5	128.33	0.22	78	57	2	73	0.4933
29	5	148.33	-0.11	-39	60	3	79	0.2648
16	4	105.33	0.44	158	137	4	147	0.4253

Sakhmet: $n_S = \mathbf{3}$, $N_S = \mathbf{105}$, $q_B = 0.0286$

$P = 2.85$, $\Theta_R = -86$ Periodicity: $z = 3.0$, $Q_z = \mathbf{0.05}$, $z_x = 0.0$								
D	M	s_i	ϕ_i	Θ_i	$\Delta\Theta_i$	n_1	n_2	Q_B
13	6	162.33	-0.23	**-82**	4	1	3	0.0833
7	10	276.33	-0.23	**-82**	4	2	4	0.0047
27	8	236.33	-0.26	**-95**	8	**3**	**6**	**0.0004**

$P = 29.60$, $\Theta_R = 78$ No periodicity								
D	M	s_i	ϕ_i	Θ_i	$\Delta\Theta_i$	n_1	n_2	Q_B
7	10	276.33	0.22	78	0	1	1	0.0286
13	6	162.33	0.37	132	54	2	32	0.2323
27	8	236.33	-0.13	-48	126	3	70	0.3233

Ennead: $n_G = \mathbf{18}$, $N_G = \mathbf{177}$, $q_B = 0.1017$

$P = 2.85$, $\Theta_R = 46$ Weak periodicity: $z = 2.2$, $Q_z = \mathbf{0.1}$, $z_x = \mathbf{1.1}$								
D	M	g_i	ϕ_i	Θ_i	$\Delta\Theta_i$	n_1	n_2	Q_B
10	3	69.33	0.14	51	4	1	3	0.2751
24	5	143.33	0.11	38	9	2	9	0.2310
18	9	257.33	0.11	38	9	3	10	0.0731
17	2	46.33	0.07	25	21	4	24	0.2231
8	8	217.33	0.07	25	21	5	27	0.1334
6	2	35.33	0.21	76	29	6	36	0.1539
30	5	149.33	0.21	76	29	7	37	0.0764
14	2	43.33	0.02	6	40	8	44	0.0735
19	1	18.33	0.25	88	42	9	48	0.0506
9	2	38.33	0.26	95	48	10	57	0.0601
16	12	345.33	-0.02	-6	53	11	62	0.0468
2	1	1.33	0.28	101	55	**12**	**63**	**0.0237**
5	4	94.33	-0.09	-32	78	13	91	0.1317
1	2	30.33	0.46	164	118	14	126	0.4052
1	4	90.33	-0.49	-177	137	15	143	0.4899
16	2	45.33	-0.28	-101	147	16	154	0.5029
13	4	102.33	-0.28	-101	147	17	155	0.4091
30	4	119.33	-0.32	-114	160	18	166	0.4238

$P = 29.60$, $\Theta_R = 64$ No periodicity								
D	M	g_i	ϕ_i	Θ_i	$\Delta\Theta_i$	n_1	n_2	Q_B
9	2	38.33	0.18	64	0	1	1	0.1017
10	3	69.33	0.22	81	17	2	19	0.5893
8	8	217.33	0.22	81	17	3	20	0.3327
6	2	35.33	0.08	27	37	4	39	0.5701
5	4	94.33	0.07	25	39	5	42	0.4267
13	4	102.33	0.34	122	58	6	60	0.5799
14	2	43.33	0.35	124	61	7	63	0.4622
16	2	45.33	0.41	149	85	8	91	0.7184
1	4	90.33	-0.07	-24	88	9	96	0.6511
2	1	1.33	-0.07	-26	90	10	99	0.5577
30	5	149.33	-0.07	-26	90	11	100	0.4392
30	4	119.33	-0.09	-31	95	12	105	0.3801
17	2	46.33	0.45	161	97	13	106	0.2802
1	2	30.33	-0.09	-34	97	14	108	0.2069
19	1	18.33	-0.50	-180	117	15	126	0.2993
16	12	345.33	-0.45	-163	134	16	144	0.3934
18	9	257.33	-0.42	-153	143	17	150	0.3564
24	5	143.33	-0.28	-99	163	18	165	0.4135

Ennead: $n_S = 3$, $N_S = 105$, $q_B = 0.0286$

$P = 2.85$, $\Theta_R = -117$ No periodicity								
D	M	s_i	ϕ_i	Θ_i	$\Delta\Theta_i$	n_1	n_2	Q_B
15	12	344.33	-0.37	-133	16	1	16	0.3711
7	2	36.33	-0.44	-158	41	2	32	0.2323
19	10	288.33	-0.02	-6	110	3	66	0.2920

$P = 29.60$, $\Theta_R = -171$ No periodicity								
D	M	s_i	ϕ_i	Θ_i	$\Delta\Theta_i$	n_1	n_2	Q_B
15	12	344.33	-0.49	-175	4	1	7	0.1837
19	10	288.33	-0.38	-136	35	2	29	0.2006
7	2	36.33	0.11	39	150	3	98	0.5333

Heliopolis: $n_G = 4$, $N_G = 177$, $q_B = 0.0226$

$P = 2.85$, $\Theta_R = -70$ Weak periodicity: $z = 2.1$, $Q_z = 0.1$, $z_x = \mathbf{0.2}$								
D	M	g_i	ϕ_i	Θ_i	$\Delta\Theta_i$	n_1	n_2	Q_B
26	3	85.33	-0.25	-88	18	1	14	0.2739
9	7	188.33	-0.11	-38	32	2	34	0.1789
19	8	228.33	-0.07	-25	45	3	47	0.0900
10	2	39.33	-0.39	-139	69	4	69	0.0711

$P = 29.60$, $\Theta_R = 138$ No periodicity								
D	M	g_i	ϕ_i	Θ_i	$\Delta\Theta_i$	n_1	n_2	Q_B
9	7	188.33	0.24	88	50	1	44	0.6342
10	2	39.33	0.21	76	62	2	53	0.3374
19	8	228.33	-0.40	-146	76	3	67	0.1933
26	3	85.33	-0.24	-85	137	4	121	0.2928

Heliopolis: $n_S = 4$, $N_S = 105$, $q_B = 0.0381$

$P = 2.85$, $\Theta_R = -168$ Weak periodicity: $z = 2.3$, $Q_z = 0.1$, $z_x = -2.2$								
D	M	s_i	ϕ_i	Θ_i	$\Delta\Theta_i$	n_1	n_2	Q_B
22	11	321.33	-0.44	-158	10	1	8	0.2671
16	11	315.33	0.46	164	27	2	18	0.1487
3	12	332.33	0.42	152	40	3	27	0.0820
10	6	159.33	-0.28	-101	67	4	45	0.0915

$P = 29.60$, $\Theta_R = 132$ No periodicity								
D	M	s_i	ϕ_i	Θ_i	$\Delta\Theta_i$	n_1	n_2	Q_B
10	6	159.33	0.26	95	37	1	22	0.5745
16	11	315.33	-0.47	-167	61	2	41	0.4662
3	12	332.33	0.11	39	93	3	64	0.4424
22	11	321.33	-0.26	-95	134	4	85	0.4070

Enemy: $n_G = 6$, $N_G = 177$, $q_B = 0.0339$

$P = 2.85$, $\Theta_R = 140$ Weak periodicity: $z = 1.7$, $Q_z = 0.2$, $z_x = \mathbf{-1.0}$								
D	M	g_i	ϕ_i	Θ_i	$\Delta\Theta_i$	n_1	n_2	Q_B
23	10	292.33	0.39	139	1	1	2	0.0666
9	4	98.33	0.32	114	27	2	19	0.1345
9	2	38.33	0.26	95	46	3	36	0.1219
19	1	18.33	0.25	88	52	4	42	0.0533
1	8	210.33	-0.39	-139	81	5	66	0.0732
13	2	42.33	-0.33	-120	100	6	77	0.0467

$P = 29.60$, $\Theta_R = 77$ No periodicity								
D	M	g_i	ϕ_i	Θ_i	$\Delta\Theta_i$	n_1	n_2	Q_B
9	4	98.33	0.20	73	3	1	4	0.1289
9	2	38.33	0.18	64	13	2	16	0.1007
13	2	42.33	0.31	112	35	3	36	0.1219
1	8	210.33	-0.01	-5	81	4	81	0.2950
19	1	18.33	-0.50	-180	104	5	111	0.3241
23	10	292.33	-0.24	-87	164	6	164	0.4829

Enemy: $n_S = 5$, $N_S = 105$, $q_B = 0.0476$

$P = 2.85$, $\Theta_R = -121$ No periodicity								
D	M	s_i	ϕ_i	Θ_i	$\Delta\Theta_i$	n_1	n_2	Q_B
23	1	22.33	-0.35	-126	5	1	2	0.0930
26	1	25.33	-0.30	-107	14	2	10	0.0791
14	3	73.33	-0.46	-164	43	3	32	0.1941
25	8	234.33	0.04	13	134	4	82	0.5521
13	3	72.33	0.19	69	170	5	96	0.4840

$P = 29.60$, $\Theta_R = -146$ No periodicity								
D	M	s_i	ϕ_i	Θ_i	$\Delta\Theta_i$	n_1	n_2	Q_B
23	1	22.33	-0.36	-131	15	1	12	0.4432
26	1	25.33	-0.26	-95	51	2	40	0.5739
25	8	234.33	-0.20	-73	73	3	55	0.4901
14	3	73.33	0.36	129	85	4	61	0.3309
13	3	72.33	0.33	117	97	5	68	0.2228

Earth: $n_G = \mathbf{19}$, $N_G = 177$, $q_B = 0.1073$

$P = 2.85$, $\Theta_R = -61$ No periodicity								
D	M	g_i	ϕ_i	Θ_i	$\Delta\Theta_i$	n_1	n_2	Q_B
2	4	91.33	-0.14	-51	10	1	9	0.6401
29	5	148.33	-0.14	-51	10	2	10	0.2924
26	3	85.33	-0.25	-88	28	3	25	0.5119
2	6	151.33	-0.09	-32	29	4	30	0.4046
6	3	65.33	-0.26	-95	34	5	34	0.2989
1	1	0.33	-0.07	-25	36	6	38	0.2186
1	10	270.33	-0.33	-120	59	7	67	0.5884
30	2	59.33	-0.37	-133	72	8	76	0.5780
21	5	140.33	0.05	19	80	9	86	0.5835
12	12	341.33	-0.42	-152	91	10	95	0.5750
1	7	180.33	0.09	32	92	11	99	0.4993
1	6	150.33	-0.44	-158	97	12	104	0.4413
27	3	86.33	0.11	38	99	13	108	0.3748
24	5	143.33	0.11	38	99	14	109	0.2794
28	5	147.33	-0.49	-177	116	15	125	0.3645
1	11	300.33	0.19	69	130	16	137	0.4000
28	3	87.33	0.46	164	135	17	140	0.3330
7	9	246.33	0.25	88	149	18	153	0.3774
3	10	272.33	0.37	133	167	19	170	0.4623

$P = 29.60$, $\Theta_R = -27$ Periodicity: $z = 6.6$, $Q_z = \mathbf{0.001}$, $z_x = \mathbf{5.3}$								
D	M	g_i	ϕ_i	Θ_i	$\Delta\Theta_i$	n_1	n_2	Q_B
1	1	0.33	-0.11	-39	12	1	17	0.8549
29	5	148.33	-0.11	-39	12	2	18	0.5902
1	6	150.33	-0.04	-14	13	3	19	0.3344
30	2	59.33	-0.11	-41	14	4	21	0.1824
2	4	91.33	-0.03	-12	15	5	23	0.0930
1	7	180.33	-0.03	-9	17	6	26	0.0535
28	5	147.33	-0.14	-51	24	7	37	0.0957
2	6	151.33	-0.01	-2	25	8	38	0.0457
1	10	270.33	0.01	5	32	9	47	0.0599
28	3	87.33	-0.17	-60	34	10	48	0.0292
1	11	300.33	0.03	10	37	11	54	0.0266
27	3	86.33	-0.20	-73	46	12	63	0.0343
3	10	272.33	0.08	30	56	13	74	0.0506
26	3	85.33	-0.24	-85	58	14	75	0.0275
6	3	65.33	0.09	32	59	15	76	0.0143
24	5	143.33	-0.28	-99	73	16	88	0.0244
7	9	246.33	0.20	73	100	17	115	0.1084
21	5	140.33	-0.38	-136	109	18	122	0.1023
12	12	341.33	0.41	149	176	19	174	0.5045

Earth: $n_S = \mathbf{5}$, $N_S = 105$, $q_B = 0.0476$

$P = 2.85$, $\Theta_R = -75$ Weak periodicity: $z = 2.9$, $Q_z = \mathbf{0.06}$, $z_x = 0.2$								
D	M	s_i	ϕ_i	Θ_i	$\Delta\Theta_i$	n_1	n_2	Q_B
26	5	145.33	-0.19	-69	5	1	4	0.1773
27	12	356.33	-0.16	-57	18	2	12	0.1091
20	5	139.33	-0.30	-107	33	3	23	0.0941
17	5	136.33	-0.35	-126	52	4	33	0.0702
19	10	288.33	-0.02	-6	68	5	42	0.0483

$P = 29.60$, $\Theta_R = -119$ Weak periodicity: $z = 1.8$, $Q_z = \mathbf{0.2}$, $z_x = -0.4$								
D	M	s_i	ϕ_i	Θ_i	$\Delta\Theta_i$	n_1	n_2	Q_B
19	10	288.33	-0.38	-136	17	1	12	0.4432
20	5	139.33	-0.41	-148	29	2	23	0.3000
26	5	145.33	-0.21	-75	44	3	33	0.2065
17	5	136.33	0.49	175	66	4	49	0.2040
27	12	356.33	-0.08	-29	90	5	62	0.1725

Heaven: $n_G = \mathbf{19}$, $N_G = \mathbf{177}$, $q_B = 0.1073$

$P = 2.85$, $\Theta_R = 10$ No periodicity								
D	M	g_i	ϕ_i	Θ_i	$\Delta\Theta_i$	n_1	n_2	Q_B
2	12	331.33	0.07	25	15	1	22	0.9178
1	3	60.33	-0.02	-6	16	2	23	0.7236
1	7	180.33	0.09	32	22	3	29	0.6153
24	5	143.33	0.11	38	28	4	41	0.6547
19	8	228.33	-0.07	-25	35	5	53	0.6854
10	3	69.33	0.14	51	41	6	56	0.5654
1	11	300.33	0.19	69	59	7	78	0.7449
2	4	91.33	-0.14	-51	61	8	80	0.6372
6	2	35.33	0.21	76	66	9	83	0.5393
19	1	18.33	0.25	88	78	10	92	0.5328
2	2	31.33	-0.19	-69	79	11	97	0.4713
26	3	85.33	-0.25	-88	98	12	115	0.5857
5	1	4.33	0.33	120	110	13	125	0.5907
13	4	102.33	-0.28	-101	111	14	128	0.5123
1	12	330.33	-0.28	-101	111	15	130	0.4244
3	10	272.33	0.37	133	123	16	132	0.3425
1	8	210.33	-0.39	-139	149	17	153	0.4783
1	6	150.33	-0.44	-158	168	18	166	0.5188
15	8	224.33	-0.47	-171	179	19	175	0.5150

$P = 29.60$, $\Theta_R = 0$ Periodicity: $z = 3.4$, $Q_z = \mathbf{0.03}$, $z_x = \mathbf{3.4}$								
D	M	g_i	ϕ_i	Θ_i	$\Delta\Theta_i$	n_1	n_2	Q_B
1	8	210.33	-0.01	-5	4	1	7	0.5484
1	7	180.33	-0.03	-9	9	2	16	0.5247
5	1	4.33	0.03	10	10	3	18	0.3036
1	11	300.33	0.03	10	10	4	19	0.1396
2	4	91.33	-0.03	-12	12	5	20	0.0559
1	6	150.33	-0.04	-14	14	6	23	0.0309
1	12	330.33	0.04	15	15	7	25	0.0138
2	2	31.33	-0.06	-22	22	8	31	0.0144
6	2	35.33	0.08	27	27	9	41	0.0275
2	12	331.33	0.08	27	27	10	42	0.0118
1	3	60.33	-0.08	-29	29	11	43	0.0048
3	10	272.33	0.08	30	30	**12**	**45**	**0.0022**
10	3	69.33	0.22	81	81	13	99	0.2628
26	3	85.33	-0.24	-85	85	14	104	0.2238
24	5	143.33	-0.28	-99	99	15	117	0.2724
13	4	102.33	0.34	122	122	16	136	0.3884
19	8	228.33	-0.40	-146	146	17	150	0.4446
15	8	224.33	0.46	166	166	18	167	0.5294
19	1	18.33	-0.50	-180	180	19	175	0.5150

Heaven: $n_S = \mathbf{4}$, $N_S = 105$, $q_B = 0.0381$

$P = 2.85$, $\Theta_R = 103$ No periodicity								
D	M	s_i	ϕ_i	Θ_i	$\Delta\Theta_i$	n_1	n_2	Q_B
22	1	21.33	0.30	107	5	1	2	0.0747
20	6	169.33	0.23	82	20	2	13	0.0857
19	5	138.33	0.35	126	24	3	16	0.0213
26	5	145.33	-0.19	-69	172	4	99	0.5237

$P = 29.60$, $\Theta_R = -132$ Weak periodicity: $z = 2.9$, $Q_z = \mathbf{0.06}$, $z_x = -1.3$								
D	M	s_i	ϕ_i	Θ_i	$\Delta\Theta_i$	n_1	n_2	Q_B
22	1	21.33	-0.40	-143	11	1	7	0.2381
20	6	169.33	-0.40	-143	11	2	8	0.0349
19	5	138.33	-0.44	-160	28	3	20	0.0388
26	5	145.33	-0.21	-75	57	4	48	0.1096

Busiris: $n_G = \mathbf{4}$, $N_G = 177$, $q_B = 0.0226$

$P = 2.85$, $\Theta_R = 19$ No periodicity								
D	M	g_i	ϕ_i	Θ_i	$\Delta\Theta_i$	n_1	n_2	Q_B
2	8	211.33	-0.04	-13	32	1	44	0.6342
28	10	297.33	0.14	51	32	2	47	0.2873
6	7	185.33	-0.16	-57	76	3	94	0.3573
30	7	209.33	0.26	95	76	4	95	0.1684

$P = 29.60$, $\Theta_R = 3$ Periodicity: $z = 3.0$, $Q_z = \mathbf{0.05}$, $z_x = \mathbf{3.0}$								
D	M	g_i	ϕ_i	Θ_i	$\Delta\Theta_i$	n_1	n_2	Q_B
2	8	211.33	0.02	8	5	1	6	0.1282
30	7	209.33	-0.05	-17	20	2	29	0.1391
28	10	297.33	-0.07	-26	29	3	45	0.0813
6	7	185.33	0.14	51	48	4	63	0.0543

Busiris: $n_S = 3$, $N_S = 105$, $q_B = 0.0286$

$P = 2.85$, $\Theta_R = -123$ No periodicity								
D	M	s_i	ϕ_i	Θ_i	$\Delta\Theta_i$	n_1	n_2	Q_B
14	5	133.33	-0.40	-145	22	1	19	0.4235
26	5	145.33	-0.19	-69	53	2	39	0.3068
26	2	55.33	0.23	82	155	3	87	0.4543

$P = 29.60$, $\Theta_R = -111$ No periodicity								
D	M	s_i	ϕ_i	Θ_i	$\Delta\Theta_i$	n_1	n_2	Q_B
26	2	55.33	-0.25	-90	21	1	16	0.3711
26	5	145.33	-0.21	-75	35	2	27	0.1798
14	5	133.33	0.39	139	110	3	68	0.3076

Rebel: $n_G = \mathbf{3}$, $N_G = 177$, $q_B = 0.0169$

$P = 2.85$, $\Theta_R = 125$								
No periodicity								
D	M	g_i	ϕ_i	Θ_i	$\Delta\Theta_i$	n_1	n_2	Q_B
2	1	1.33	0.28	101	24	1	16	0.2393
7	9	246.33	0.25	88	36	2	30	0.0915
1	8	210.33	-0.39	-139	96	3	87	0.1837

$P = 29.60$, $\Theta_R = 11$								
Weak periodicity: $z = 1.6$, $Q_z = \mathbf{0.2}$, $z_x = \mathbf{1.6}$								
D	M	g_i	ϕ_i	Θ_i	$\Delta\Theta_i$	n_1	n_2	Q_B
1	8	210.33	-0.01	-5	16	1	20	0.2896
2	1	1.33	-0.07	-26	38	2	49	0.2017
7	9	246.33	0.20	73	62	3	81	0.1587

Rebel: $n_S = 3$, $N_S = 105$, $q_B = 0.0286$

$P = 2.85$, $\Theta_R = 107$								
No periodicity								
D	M	s_i	ϕ_i	Θ_i	$\Delta\Theta_i$	n_1	n_2	Q_B
12	2	41.33	0.32	114	7	1	5	0.1349
23	12	352.33	0.44	158	51	2	38	0.2962
20	2	49.33	0.12	44	63	3	42	0.1180

$P = 29.60$, $\Theta_R = -162$								
No periodicity								
D	M	s_i	ϕ_i	Θ_i	$\Delta\Theta_i$	n_1	n_2	Q_B
20	2	49.33	-0.45	-163	0	1	1	0.0286
23	12	352.33	-0.22	-77	85	2	63	0.5406
12	2	41.33	0.28	100	98	3	69	0.3155

Thoth: $n_G = 10$, $N_G = 177$, $q_B = 0.0565$

$P = 2.85$, $\Theta_R = 115$								
No periodicity								
D	M	g_i	ϕ_i	Θ_i	$\Delta\Theta_i$	n_1	n_2	Q_B
9	4	98.33	0.32	114	1	1	1	0.0565
13	7	192.33	0.30	107	8	2	6	0.0411
26	4	115.33	0.28	101	14	3	9	0.0117
19	9	258.33	0.46	164	49	4	41	0.1998
3	2	32.33	0.16	57	58	5	45	0.1087
1	4	90.33	-0.49	-177	68	6	56	0.0953
28	5	147.33	-0.49	-177	68	7	57	0.0409
27	3	86.33	0.11	38	77	8	65	0.0297
29	5	148.33	-0.14	-51	166	9	165	0.5908
30	10	299.33	-0.16	-57	172	10	173	0.5164

$P = 29.60$, $\Theta_R = -39$								
Weak periodicity: $z = 2.1$, $Q_z = 0.1$, $z_x = \mathbf{1.3}$								
D	M	g_i	ϕ_i	Θ_i	$\Delta\Theta_i$	n_1	n_2	Q_B
29	5	148.33	-0.11	-39	1	1	2	0.1098
28	5	147.33	-0.14	-51	11	2	15	0.2066
1	4	90.33	-0.07	-24	15	3	21	0.1124
3	2	32.33	-0.03	-9	30	4	39	0.1766
27	3	86.33	-0.20	-73	33	5	46	0.1166
30	10	299.33	-0.01	-2	37	6	52	0.0720
26	4	115.33	-0.22	-80	41	7	54	0.0317
19	9	258.33	-0.39	-141	101	8	110	0.2827
9	4	98.33	0.20	73	113	9	120	0.2381
13	7	192.33	0.38	137	176	10	176	0.5384

Thoth: $n_S = 2$, $N_S = 105$, $q_B = 0.0190$

$P = 2.85$, $\Theta_R = 66$								
Weak periodicity: $z = 1.7$, $Q_z = 0.2$, $z_x = 0.3$								
D	M	s_i	ϕ_i	Θ_i	$\Delta\Theta_i$	n_1	n_2	Q_B
20	2	49.33	0.12	44	22	1	18	0.2926
10	7	189.33	0.25	88	22	2	19	0.0501

$P = 29.60$, $\Theta_R = 149$								
No periodicity								
D	M	s_i	ϕ_i	Θ_i	$\Delta\Theta_i$	n_1	n_2	Q_B
20	2	49.33	-0.45	-163	49	1	34	0.4800
10	7	189.33	0.28	100	49	2	35	0.1432

Onnophris: $n_G = 7$, $N_G = 177$, $q_B = 0.0395$

$P = 2.85$, $\Theta_R = -138$								
No periodicity								
D	M	g_i	ϕ_i	Θ_i	$\Delta\Theta_i$	n_1	n_2	Q_B
28	7	207.33	-0.44	-158	20	1	14	0.4316
16	2	45.33	-0.28	-101	37	2	24	0.2451
1	12	330.33	-0.28	-101	37	3	27	0.0894
28	3	87.33	0.46	164	58	4	47	0.1144
29	8	238.33	0.44	158	64	5	54	0.0619
28	6	177.33	0.04	13	151	6	141	0.4861
24	3	83.33	0.05	19	157	7	146	0.3567

$P = 29.60$, $\Theta_R = -47$								
Weak periodicity: $z = 2.1$, $Q_z = 0.1$, $z_x = \mathbf{1.0}$								
D	M	g_i	ϕ_i	Θ_i	$\Delta\Theta_i$	n_1	n_2	Q_B
28	6	177.33	-0.13	-46	1	1	2	0.0775
28	7	207.33	-0.11	-41	6	2	7	0.0288
28	3	87.33	-0.17	-60	14	3	16	0.0235
29	8	238.33	-0.07	-24	23	4	29	0.0264
1	12	330.33	0.04	15	62	5	78	0.1959
24	3	83.33	-0.30	-109	62	6	79	0.0928
16	2	45.33	0.41	149	164	7	167	0.4918

Onnophris: $n_S = 3$, $N_S = 105$, $q_B = 0.0286$

$P = 2.85$, $\Theta_R = -32$								
No periodicity								
D	M	s_i	ϕ_i	Θ_i	$\Delta\Theta_i$	n_1	n_2	Q_B
23	11	322.33	-0.09	-32	1	1	1	0.0286
24	8	233.33	-0.32	-114	82	2	39	0.3068
13	3	72.33	0.19	69	102	3	55	0.2077

$P = 29.60$, $\Theta_R = -102$								
No periodicity								
D	M	s_i	ϕ_i	Θ_i	$\Delta\Theta_i$	n_1	n_2	Q_B
24	8	233.33	-0.24	-85	17	1	13	0.3140
23	11	322.33	-0.23	-82	20	2	15	0.0670
13	3	72.33	0.33	117	141	3	82	0.4166

Nut: $n_G = 3$, $N_G = 177$, $q_B = 0.0169$

$P = 2.85$, $\Theta_R = 55$ No periodicity								
D	M	g_i	ϕ_i	Θ_i	$\Delta\Theta_i$	n_1	n_2	Q_B
22	12	351.33	0.09	32	24	1	28	0.3804
18	7	197.33	0.05	19	37	2	44	0.1711
28	1	27.33	0.40	145	90	3	100	0.2407

$P = 29.60$, $\Theta_R = -105$ Weak periodicity: $z = 1.8$, $Q_z = 0.2$, $z_x = -\mathbf{0.1}$								
D	M	g_i	ϕ_i	Θ_i	$\Delta\Theta_i$	n_1	n_2	Q_B
22	12	351.33	-0.25	-90	16	1	13	0.1993
28	1	27.33	-0.19	-70	35	2	26	0.0714
18	7	197.33	-0.45	-163	57	3	45	0.0408

Nut: $n_S = 4$, $N_S = 105$, $q_B = 0.0381$

$P = 2.85$, $\Theta_R = 81$ No periodicity								
D	M	s_i	ϕ_i	Θ_i	$\Delta\Theta_i$	n_1	n_2	Q_B
17	8	226.33	0.23	82	1	1	3	0.1100
12	2	41.33	0.32	114	33	2	24	0.2321
20	2	49.33	0.12	44	37	3	27	0.0820
4	10	273.33	-0.28	-101	178	4	105	0.5703

$P = 29.60$, $\Theta_R = 139$ No periodicity								
D	M	s_i	ϕ_i	Θ_i	$\Delta\Theta_i$	n_1	n_2	Q_B
12	2	41.33	0.28	100	39	1	24	0.6063
17	8	226.33	-0.47	-170	51	2	34	0.3735
20	2	49.33	-0.45	-163	58	3	41	0.2046
4	10	273.33	0.12	42	98	4	66	0.2434

Heart: $n_G = 27$, $N_G = 177$, $q_B = 0.1525$

$P = 2.85$, $\Theta_R = -91$ No periodicity								
D	M	g_i	ϕ_i	Θ_i	$\Delta\Theta_i$	n_1	n_2	Q_B
9	1	8.33	-0.26	-95	4	1	4	0.4842
6	3	65.33	-0.26	-95	4	2	5	0.1695
16	2	45.33	-0.28	-101	10	3	11	0.2289
13	4	102.33	-0.28	-101	10	4	12	0.0969
7	8	216.33	-0.28	-101	10	5	13	0.0365
29	3	88.33	-0.19	-69	22	6	19	0.0576
30	4	119.33	-0.32	-114	23	7	22	0.0399
13	2	42.33	-0.33	-120	29	8	24	0.0218
9	5	128.33	-0.16	-57	34	9	29	0.0247
29	5	148.33	-0.14	-51	41	10	34	0.0267
10	2	39.33	-0.39	-139	48	11	41	0.0396
4	6	153.33	-0.39	-139	48	12	42	0.0201
12	12	341.33	-0.42	-152	60	13	54	0.0594
4	4	93.33	-0.44	-158	67	14	61	0.0729
16	12	345.33	-0.02	-6	85	15	83	0.2791
24	1	23.33	0.00	0	91	16	87	0.2470
9	11	308.33	0.00	0	91	17	90	0.2048
9	10	278.33	0.47	171	98	18	99	0.2457
28	3	87.33	0.46	164	105	19	105	0.2447
24	3	83.33	0.05	19	110	20	109	0.2182
14	4	103.33	0.07	25	116	21	115	0.2178
25	10	294.33	0.09	32	123	22	126	0.2795
25	3	84.33	0.40	145	124	23	129	0.2401
25	12	354.33	0.14	51	142	24	147	0.3929
2	1	1.33	0.28	101	168	25	164	0.5347
9	2	38.33	0.26	95	174	26	170	0.5269
7	9	246.33	0.25	88	180	27	176	0.5193

$P = 29.60$, $\Theta_R = 47$ No periodicity								
D	M	g_i	ϕ_i	Θ_i	$\Delta\Theta_i$	n_1	n_2	Q_B
9	1	8.33	0.16	59	11	1	8	0.7340
6	3	65.33	0.09	32	15	2	14	0.6531
9	2	38.33	0.18	64	16	3	16	0.4502
7	8	216.33	0.19	68	21	4	22	0.4383
4	6	153.33	0.06	22	25	5	27	0.3959
7	9	246.33	0.20	73	26	6	30	0.3034
10	2	39.33	0.21	76	29	7	31	0.1841
9	5	128.33	0.22	78	31	8	34	0.1361
4	4	93.33	0.03	13	35	9	38	0.1145
9	10	278.33	0.28	103	55	10	64	0.5202
9	11	308.33	0.30	107	60	11	71	0.5281
13	2	42.33	0.31	112	65	12	76	0.4969
2	1	1.33	-0.07	-26	74	13	86	0.5601
13	4	102.33	0.34	122	75	14	89	0.4952
30	4	119.33	-0.09	-31	79	15	95	0.4857
29	5	148.33	-0.11	-39	86	16	101	0.4769
14	4	103.33	0.37	134	87	17	102	0.3864
29	3	88.33	-0.13	-48	96	18	110	0.4127
25	12	354.33	-0.15	-53	100	19	113	0.3605
16	2	45.33	0.41	149	101	20	114	0.2842
12	12	341.33	0.41	149	101	21	115	0.2178
28	3	87.33	-0.17	-60	108	22	120	0.2056
25	10	294.33	-0.17	-63	110	23	122	0.1631
25	3	84.33	-0.27	-97	144	24	154	0.4888
16	12	345.33	-0.45	-163	150	25	160	0.4816
24	3	83.33	-0.30	-109	156	26	164	0.4484
24	1	23.33	-0.33	-119	166	27	169	0.4293

Heart: $n_S = \mathbf{5}$, $N_S = \mathbf{105}$, $q_B = 0.0476$

$P = 2.85$, $\Theta_R = -172$ Periodicity: $z = 3.2$, $Q_z = \mathbf{0.04}$, $z_x = -\mathbf{3.1}$								
D	M	s_i	ϕ_i	Θ_i	$\Delta\Theta_i$	n_1	n_2	Q_B
14	3	73.33	-0.46	-164	8	1	5	0.2165
22	7	201.33	0.46	164	24	2	14	0.1414
5	11	304.33	-0.40	-145	26	3	18	0.0516
23	1	22.33	-0.35	-126	45	4	28	0.0423
10	11	309.33	0.35	126	62	**5**	**39**	**0.0367**

$P = 29.60$, $\Theta_R = 154$ No periodicity								
D	M	s_i	ϕ_i	Θ_i	$\Delta\Theta_i$	n_1	n_2	Q_B
14	3	73.33	0.36	129	24	1	15	0.5190
10	11	309.33	0.33	120	34	2	22	0.2821
23	1	22.33	-0.36	-131	75	3	54	0.4779
22	7	201.33	-0.32	-114	92	4	65	0.3746
5	11	304.33	0.16	59	95	5	66	0.2055

Nun: $n_G = 7$, $N_G = 177$, $q_B = 0.0395$

$P = 2.85$, $\Theta_R = -38$ No periodicity								
D	M	g_i	ϕ_i	Θ_i	$\Delta\Theta_i$	n_1	n_2	Q_B
1	1	0.33	-0.07	-25	13	1	14	0.4316
12	7	191.33	-0.05	-19	19	2	25	0.2599
19	2	48.33	-0.23	-82	44	3	55	0.3715
17	2	46.33	0.07	25	63	4	73	0.3269
30	2	59.33	-0.37	-133	95	5	111	0.4484
30	5	149.33	0.21	76	114	6	121	0.3456
1	4	90.33	-0.49	-177	139	7	148	0.3696

$P = 29.60$, $\Theta_R = -52$ No periodicity								
D	M	g_i	ϕ_i	Θ_i	$\Delta\Theta_i$	n_1	n_2	Q_B
30	2	59.33	-0.11	-41	11	1	10	0.3320
1	1	0.33	-0.11	-39	13	2	14	0.1039
30	5	149.33	-0.07	-26	25	3	30	0.1140
1	4	90.33	-0.07	-24	28	4	32	0.0364
19	2	48.33	-0.49	-175	123	5	127	0.5671
17	2	46.33	0.45	161	147	6	151	0.5529
12	7	191.33	0.35	124	176	7	175	0.5412

Nun: $n_S =$ **3**, $N_S =$ **105**, $q_B = 0.0286$

$P = 2.85$, $\Theta_R = -122$ Weak periodicity: $z = 2.9$, $Q_z =$ **0.06**, $z_x = -0.8$								
D	M	s_i	ϕ_i	Θ_i	$\Delta\Theta_i$	n_1	n_2	Q_B
17	5	136.33	-0.35	-126	4	1	4	0.1095
15	12	344.33	-0.37	-133	10	2	9	0.0257
23	3	82.33	-0.30	-107	15	**3**	**11**	**0.0032**

$P = 29.60$, $\Theta_R = -161$ Weak periodicity: $z = 2.4$, $Q_z = 0.1$, $z_x = -2.1$								
D	M	s_i	ϕ_i	Θ_i	$\Delta\Theta_i$	n_1	n_2	Q_B
15	12	344.33	-0.49	-175	14	1	15	0.3526
17	5	136.33	0.49	175	24	2	22	0.1295
23	3	82.33	-0.34	-121	40	3	33	0.0674

Seth: $n_G = 5$, $N_G = 177$, $q_B = 0.0282$

$P = 2.85$, $\Theta_R = 11$ No periodicity								
D	M	g_i	ϕ_i	Θ_i	$\Delta\Theta_i$	n_1	n_2	Q_B
27	1	26.33	0.05	19	8	1	9	0.2273
27	3	86.33	0.11	38	27	2	40	0.3126
13	12	342.33	-0.07	-25	36	3	55	0.2031
29	3	88.33	-0.19	-69	80	4	98	0.3003
9	4	98.33	0.32	114	103	5	118	0.2417

$P = 29.60$, $\Theta_R = -73$ No periodicity								
D	M	g_i	ϕ_i	Θ_i	$\Delta\Theta_i$	n_1	n_2	Q_B
27	3	86.33	-0.20	-73	0	1	1	0.0282
27	1	26.33	-0.23	-82	10	2	9	0.0252
29	3	88.33	-0.13	-48	24	3	24	0.0293
13	12	342.33	0.45	161	126	4	128	0.4900
9	4	98.33	0.20	73	146	5	146	0.3958

Seth: $n_S =$ **9**, $N_S = 105$, $q_B = 0.0857$

$P = 2.85$, $\Theta_R = 57$ No periodicity								
D	M	s_i	ϕ_i	Θ_i	$\Delta\Theta_i$	n_1	n_2	Q_B
13	3	72.33	0.19	69	12	1	8	0.5117
20	2	49.33	0.12	44	13	2	12	0.2750
17	8	226.33	0.23	82	25	3	18	0.1964
11	12	340.33	0.23	82	25	4	19	0.0739
12	2	41.33	0.32	114	56	5	29	0.0977
18	3	77.33	-0.05	-19	76	6	43	0.1593
26	1	25.33	-0.30	-107	165	7	92	0.6831
17	7	196.33	-0.30	-107	165	8	95	0.5739
24	8	233.33	-0.32	-114	171	9	100	0.4902

$P = 29.60$, $\Theta_R = -180$ Periodicity: $z = 3.1$, $Q_z =$ **0.05**, $z_x = -3.1$								
D	M	s_i	ϕ_i	Θ_i	$\Delta\Theta_i$	n_1	n_2	Q_B
18	3	77.33	0.49	178	2	1	1	0.0857
17	7	196.33	-0.49	**-175**	6	2	5	0.0617
17	8	226.33	-0.47	-170	11	3	10	0.0478
20	2	49.33	-0.45	-163	18	4	16	0.0425
11	12	340.33	0.38	137	43	5	33	0.1483
13	3	72.33	0.33	117	62	6	47	0.2122
12	2	41.33	0.28	100	79	7	56	0.2014
26	1	25.33	-0.26	-95	86	8	62	0.1591
24	8	233.33	-0.24	-85	96	9	68	0.1260

Osiris: $n_G = 8$, $N_G = 177$, $q_B = 0.0452$

$P = 2.85$, $\Theta_R = -82$								
No periodicity								
D	M	g_i	ϕ_i	Θ_i	$\Delta\Theta_i$	n_1	n_2	Q_B
16	2	45.33	-0.28	-101	19	1	18	0.5650
6	7	185.33	-0.16	-57	25	2	23	0.2791
1	10	270.33	-0.33	-120	38	3	39	0.2579
13	12	342.33	-0.07	-25	57	4	55	0.2368
28	7	207.33	-0.44	-158	76	5	76	0.2598
11	4	100.33	0.02	6	88	6	86	0.1935
28	3	87.33	0.46	164	114	7	122	0.3144
17	6	166.33	0.18	63	145	8	148	0.3540

$P = 29.60$, $\Theta_R = 93$								
No periodicity								
D	M	g_i	ϕ_i	Θ_i	$\Delta\Theta_i$	n_1	n_2	Q_B
11	4	100.33	0.27	98	4	1	3	0.1296
6	7	185.33	0.14	51	42	2	43	0.5846
16	2	45.33	0.41	149	56	3	50	0.3949
13	12	342.33	0.45	161	68	4	64	0.3281
17	6	166.33	-0.50	-180	87	5	83	0.3216
1	10	270.33	0.01	5	88	6	86	0.1935
28	7	207.33	-0.11	-41	134	7	137	0.4252
28	3	87.33	-0.17	-60	154	8	151	0.3746

Osiris: $n_S = \mathbf{4}$, $N_S = \mathbf{105}$, $q_B = 0.0381$

$P = 2.85$, $\Theta_R = -141$								
No periodicity								
D	M	s_i	ϕ_i	Θ_i	$\Delta\Theta_i$	n_1	n_2	Q_B
14	5	133.33	-0.40	-145	4	1	3	0.1100
14	3	73.33	-0.46	-164	23	2	18	0.1487
19	4	108.33	-0.18	-63	78	3	52	0.3180
13	3	72.33	0.19	69	150	4	92	0.4665

$P = 29.60$, $\Theta_R = 144$								
Periodicity: $z = 3.0$, $Q_z = \mathbf{0.05}$, $z_x = -2.0$								
D	M	s_i	ϕ_i	Θ_i	$\Delta\Theta_i$	n_1	n_2	Q_B
14	5	133.33	0.39	139	5	1	4	0.1439
14	3	73.33	0.36	129	14	2	9	0.0437
13	3	72.33	0.33	117	27	**3**	**15**	**0.0178**
19	4	108.33	-0.46	-165	51	4	37	0.0513

Abydos: $n_G = \mathbf{3}$, $N_G = 177$, $q_B = 0.0169$

$P = 2.85$, $\Theta_R = -14$								
No periodicity								
D	M	g_i	ϕ_i	Θ_i	$\Delta\Theta_i$	n_1	n_2	Q_B
18	5	137.33	0.00	0	14	1	22	0.3135
11	4	100.33	0.02	6	20	2	30	0.0915
28	7	207.33	-0.44	-158	144	3	146	0.4507

$P = 29.60$, $\Theta_R = 151$								
No periodicity								
D	M	g_i	ϕ_i	Θ_i	$\Delta\Theta_i$	n_1	n_2	Q_B
18	5	137.33	-0.48	-172	36	1	29	0.3909
11	4	100.33	0.27	98	54	2	43	0.1650
28	7	207.33	-0.11	-41	168	3	159	0.5066

Abydos: $n_S = \mathbf{2}$, $N_S = 105$, $q_B = 0.0190$

$P = 2.85$, $\Theta_R = 3$								
No periodicity								
D	M	s_i	ϕ_i	Θ_i	$\Delta\Theta_i$	n_1	n_2	Q_B
13	3	72.33	0.19	69	66	1	26	0.3935
13	8	222.33	-0.18	-63	66	2	30	0.1112

$P = 29.60$, $\Theta_R = 129$								
Weak periodicity: $z = 1.9$, $Q_z = 0.1$, $z_x = -0.8$								
D	M	s_i	ϕ_i	Θ_i	$\Delta\Theta_i$	n_1	n_2	Q_B
13	3	72.33	0.33	117	12	1	9	0.1589
13	8	222.33	0.39	141	12	2	12	0.0211

Lion: $n_G = 1$, $N_G = 177$, $q_B = 0.0056$

$P = 2.85$, $\Theta_R = -177$								
No periodicity								
D	M	g_i	ϕ_i	Θ_i				
1	4	90.33	-0.49	-177				

$P = 29.60$, $\Theta_R = -24$								
No periodicity								
D	M	g_i	ϕ_i	Θ_i				
1	4	90.33	-0.07	-24				

Lion: $n_S = 6$, $N_S = 105$, $q_B = 0.0571$

$P = 2.85$, $\Theta_R = -162$								
No periodicity								
D	M	s_i	ϕ_i	Θ_i	$\Delta\Theta_i$	n_1	n_2	Q_B
24	2	53.33	-0.47	-171	8	1	6	0.2975
18	10	287.33	-0.37	-133	29	2	19	0.2966
26	10	295.33	0.44	158	40	3	28	0.2135
3	8	212.33	0.32	114	84	4	56	0.3987
25	2	54.33	-0.12	-44	118	5	79	0.4735
19	10	288.33	-0.02	-6	156	6	101	0.5207

$P = 29.60$, $\Theta_R = -100$								
Weak periodicity: $z = 2.2$, $Q_z = 0.1$, $z_x = -0.1$								
D	M	s_i	ϕ_i	Θ_i	$\Delta\Theta_i$	n_1	n_2	Q_B
25	2	54.33	-0.28	-102	2	1	2	0.1110
24	2	53.33	-0.32	-114	14	2	9	0.0899
19	10	288.33	-0.38	-136	36	3	25	0.1692
18	10	287.33	-0.41	-148	48	4	33	0.1172
26	10	295.33	-0.14	-51	49	5	34	0.0425
3	8	212.33	0.06	20	119	6	70	0.2102

Man: $n_G = 5$, $N_G = 177$, $q_B = 0.0282$

$P = 2.85$, $\Theta_R = -27$ No periodicity								
D	M	g_i	ϕ_i	Θ_i	$\Delta\Theta_i$	n_1	n_2	Q_B
16	10	285.33	-0.07	-25	2	1	5	0.1335
6	5	125.33	-0.21	-76	49	2	60	0.5083
4	5	123.33	0.09	32	59	3	73	0.3404
3	5	122.33	-0.26	-95	68	4	88	0.2378
29	2	58.33	0.28	101	128	5	134	0.3287

$P = 29.60$, $\Theta_R = 3$ No periodicity								
D	M	g_i	ϕ_i	Θ_i	$\Delta\Theta_i$	n_1	n_2	Q_B
3	5	122.33	0.01	5	2	1	3	0.0824
4	5	123.33	0.05	17	14	2	22	0.1272
6	5	125.33	0.12	42	39	3	53	0.1886
29	2	58.33	-0.15	-53	56	4	72	0.1465
16	10	285.33	-0.48	-172	175	5	172	0.5366

Man: $n_S = \mathbf{6}$, $N_S = \mathbf{105}$, $q_B = 0.0571$

$P = 2.85$, $\Theta_R = -165$ No periodicity								
D	M	s_i	ϕ_i	Θ_i	$\Delta\Theta_i$	n_1	n_2	Q_B
8	11	307.33	-0.35	-126	39	1	29	0.8185
6	8	215.33	0.37	133	62	2	41	0.6878
4	10	273.33	-0.28	-101	64	3	43	0.4486
30	6	179.33	-0.26	-95	70	4	47	0.2806
7	11	306.33	0.30	107	88	5	60	0.2579
7	5	126.33	0.14	51	144	6	95	0.4610

$P = 29.60$, $\Theta_R = 54$ Periodicity: $z = 3.9$, $Q_z = \mathbf{0.02}$, $z_x = 1.4$								
D	M	s_i	ϕ_i	Θ_i	$\Delta\Theta_i$	n_1	n_2	Q_B
7	5	126.33	0.15	54	0	1	1	0.0571
6	8	215.33	0.16	56	2	2	2	0.0033
4	10	273.33	0.12	42	12	3	7	0.0055
7	11	306.33	0.23	83	29	4	17	0.0139
8	11	307.33	0.26	95	41	**5**	**23**	**0.0086**
30	6	179.33	-0.06	-22	76	6	35	0.0135

Flame: $n_S = \mathbf{4}$, $N_S = \mathbf{105}$, $q_B = 0.0381$

$P = 2.85$, $\Theta_R = 123$ No periodicity								
D	M	s_i	ϕ_i	Θ_i	$\Delta\Theta_i$	n_1	n_2	Q_B
11	1	10.33	0.44	158	35	1	19	0.5219
10	7	189.33	0.25	88	35	2	20	0.1759
11	5	130.33	-0.46	-164	73	3	49	0.2865
7	5	126.33	0.14	51	73	4	50	0.1224

$P = 29.60$, $\Theta_R = 85$ Periodicity: $z = 3.6$, $Q_z = \mathbf{0.03}$, $z_x = 0.0$								
D	M	s_i	ϕ_i	Θ_i	$\Delta\Theta_i$	n_1	n_2	Q_B
11	1	10.33	0.23	83	2	1	1	0.0381
10	7	189.33	0.28	100	15	2	10	0.0533
11	5	130.33	0.28	103	17	3	12	0.0094
7	5	126.33	0.15	54	31	**4**	**17**	**0.0034**

Eye: $n_G = 4$, $N_G = 177$, $q_B = 0.0226$

$P = 2.85$, $\Theta_R = -24$ No periodicity								
D	M	g_i	ϕ_i	Θ_i	$\Delta\Theta_i$	n_1	n_2	Q_B
10	12	339.33	-0.12	-44	21	1	32	0.5188
19	12	348.33	0.04	13	36	2	51	0.3208
8	8	217.33	0.07	25	49	3	63	0.1706
10	4	99.33	-0.33	-120	96	4	111	0.2430

$P = 29.60$, $\Theta_R = 115$ No periodicity								
D	M	g_i	ϕ_i	Θ_i	$\Delta\Theta_i$	n_1	n_2	Q_B
10	12	339.33	0.35	124	10	1	11	0.2223
10	4	99.33	0.24	85	29	2	24	0.1016
8	8	217.33	0.22	81	34	3	27	0.0225
19	12	348.33	-0.35	-126	119	4	110	0.2381

Eye: $n_S = 5$, $N_S = 105$, $q_B = 0.0476$

$P = 2.85$, $\Theta_R = 81$ No periodicity								
D	M	s_i	ϕ_i	Θ_i	$\Delta\Theta_i$	n_1	n_2	Q_B
7	7	186.33	0.19	69	11	1	11	0.4153
20	4	109.33	0.18	63	18	2	14	0.1414
3	8	212.33	0.32	114	33	3	25	0.1141
14	11	313.33	-0.25	-88	169	4	96	0.6759
11	11	310.33	-0.30	-107	172	5	101	0.5291

$P = 29.60$, $\Theta_R = 124$ No periodicity								
D	M	s_i	ϕ_i	Θ_i	$\Delta\Theta_i$	n_1	n_2	Q_B
11	11	310.33	0.37	132	8	1	8	0.3232
14	11	313.33	0.47	168	45	2	26	0.3531
7	7	186.33	0.18	64	60	3	36	0.2446
20	4	109.33	-0.42	-153	84	4	55	0.2660
3	8	212.33	0.06	20	104	5	68	0.2228

Fire: $n_G = 4$, $N_G = 177$, $q_B = 0.0226$

$P = 2.85$, $\Theta_R = 122$ No periodicity								
D	M	g_i	ϕ_i	Θ_i	$\Delta\Theta_i$	n_1	n_2	Q_B
19	1	18.33	0.25	88	33	1	25	0.4353
29	8	238.33	0.44	158	36	2	31	0.1548
30	5	149.33	0.21	76	46	3	38	0.0542
7	8	216.33	-0.28	-101	137	4	129	0.3336

$P = 29.60$, $\Theta_R = 4$ No periodicity								
D	M	g_i	ϕ_i	Θ_i	$\Delta\Theta_i$	n_1	n_2	Q_B
29	8	238.33	-0.07	-24	28	1	40	0.5992
30	5	149.33	-0.07	-26	30	2	44	0.2621
7	8	216.33	0.19	68	65	3	81	0.2770
19	1	18.33	-0.50	-180	177	4	173	0.5508

Fire: $n_S = 7$, $N_S = 105$, $q_B = 0.0667$

$P = 2.85$, $\Theta_R = 171$ No periodicity								
D	M	s_i	ϕ_i	Θ_i	$\Delta\Theta_i$	n_1	n_2	Q_B
11	1	10.33	0.44	158	13	1	6	0.3390
29	4	118.33	0.33	120	51	2	30	0.6033
7	11	306.33	0.30	107	63	3	41	0.5207
23	1	22.33	-0.35	-126	63	4	42	0.3064
23	3	82.33	-0.30	-107	82	5	52	0.2647
10	5	129.33	0.19	69	101	6	68	0.2999
14	11	313.33	-0.25	-88	101	7	72	0.2033

$P = 29.60$, $\Theta_R = 143$ No periodicity								
D	M	s_i	ϕ_i	Θ_i	$\Delta\Theta_i$	n_1	n_2	Q_B
14	11	313.33	0.47	168	25	1	14	0.6194
10	5	129.33	0.25	90	53	2	38	0.7301
11	1	10.33	0.23	83	60	3	43	0.5533
7	11	306.33	0.23	83	60	4	44	0.3372
23	1	22.33	-0.36	-131	86	5	58	0.3441
23	3	82.33	-0.34	-121	96	6	65	0.2649
29	4	118.33	-0.12	-43	173	7	102	0.5243

Majesty: $n_G = 13$, $N_G = 177$, $q_B = 0.0734$

$P = 2.85$, $\Theta_R = -64$ No periodicity								
D	M	g_i	ϕ_i	Θ_i	$\Delta\Theta_i$	n_1	n_2	Q_B
2	2	31.33	-0.19	-69	6	1	2	0.1415
18	1	17.33	-0.11	-38	26	2	24	0.5348
9	1	8.33	-0.26	-95	31	3	28	0.3391
5	4	94.33	-0.09	-32	32	4	34	0.2375
19	8	228.33	-0.07	-25	38	5	45	0.2333
2	8	211.33	-0.04	-13	51	6	57	0.2394
24	1	23.33	0.00	0	64	7	68	0.2312
14	2	43.33	0.02	6	70	8	73	0.1662
10	2	39.33	-0.39	-139	75	9	77	0.1111
28	7	207.33	-0.44	-158	94	10	98	0.1826
1	4	90.33	-0.49	-177	113	11	120	0.2667
25	5	144.33	0.46	164	132	12	139	0.3236
9	4	98.33	0.32	114	177	13	176	0.5327

$P = 29.60$, $\Theta_R = 10$ No periodicity								
D	M	g_i	ϕ_i	Θ_i	$\Delta\Theta_i$	n_1	n_2	Q_B
2	8	211.33	0.02	8	3	1	4	0.2630
5	4	94.33	0.07	25	15	2	17	0.3582
2	2	31.33	-0.06	-22	32	3	44	0.6364
1	4	90.33	-0.07	-24	34	4	46	0.4401
9	1	8.33	0.16	59	49	5	61	0.4676
28	7	207.33	-0.11	-41	51	6	68	0.3829
9	4	98.33	0.20	73	63	7	80	0.3729
10	2	39.33	0.21	76	66	8	84	0.2750
25	5	144.33	-0.24	-87	97	9	116	0.4823
14	2	43.33	0.35	124	114	10	130	0.4871
24	1	23.33	-0.33	-119	129	11	142	0.4725
19	8	228.33	-0.40	-146	156	12	161	0.5219
18	1	17.33	0.47	168	158	13	162	0.4119

Majesty: $n_S = 7$, $N_S = 105$, $q_B = 0.0667$

$P = 2.85$, $\Theta_R = -138$ No periodicity								
D	M	s_i	ϕ_i	Θ_i	$\Delta\Theta_i$	n_1	n_2	Q_B
26	1	25.33	-0.30	-107	31	1	21	0.7652
23	3	82.33	-0.30	-107	31	2	22	0.4364
12	8	221.33	0.47	171	52	3	36	0.4339
16	11	315.33	0.46	164	58	4	41	0.2911
11	1	10.33	0.44	158	64	5	45	0.1788
20	9	259.33	-0.19	-69	68	6	49	0.1060
5	8	214.33	0.02	6	144	7	88	0.3715

$P = 29.60$, $\Theta_R = -169$ No periodicity								
D	M	s_i	ϕ_i	Θ_i	$\Delta\Theta_i$	n_1	n_2	Q_B
16	11	315.33	-0.47	-167	2	1	5	0.2918
20	9	259.33	-0.36	-129	40	2	32	0.6388
23	3	82.33	-0.34	-121	48	3	35	0.4158
12	8	221.33	0.36	129	62	4	48	0.3990
26	1	25.33	-0.26	-95	74	5	55	0.3040
11	1	10.33	0.23	83	108	6	75	0.3840
5	8	214.33	0.12	44	147	7	94	0.4379

Shu: $n_G = 3$, $N_G = 177$, $q_B = 0.0169$

$P = 2.85$, $\Theta_R = 5$ No periodicity								
D	M	g_i	ϕ_i	Θ_i	$\Delta\Theta_i$	n_1	n_2	Q_B
21	3	80.33	0.00	0	5	1	6	0.0975
30	10	299.33	-0.16	-57	62	2	84	0.4176
16	5	135.33	0.30	107	102	3	121	0.3372

$P = 29.60$, $\Theta_R = -158$ No periodicity								
D	M	g_i	ϕ_i	Θ_i	$\Delta\Theta_i$	n_1	n_2	Q_B
21	3	80.33	-0.40	-146	13	1	7	0.1128
16	5	135.33	0.45	163	39	2	25	0.0666
30	10	299.33	-0.01	-2	156	3	151	0.4725

Shu: $n_S = 1$, $N_S = 105$, $q_B = 0.0095$

$P = 2.85$, $\Theta_R = 13$ No periodicity								
D	M	s_i	ϕ_i	Θ_i				
22	10	291.33	0.04	13				

$P = 29.60$, $\Theta_R = -99$ No periodicity								
D	M	s_i	ϕ_i	Θ_i				
22	10	291.33	-0.28	-99				

Sobek: $n_G = 1$, $N_G = 177$, $q_B = 0.0056$

$P = 2.85$, $\Theta_R = 25$ No periodicity								
D	M	g_i	ϕ_i	Θ_i				
11	6	160.33	0.07	25				

$P = 29.60$, $\Theta_R = 107$ No periodicity								
D	M	g_i	ϕ_i	Θ_i				
11	6	160.33	0.30	107				

Sobek: $n_S = 3$, $N_S = 105$, $q_B = 0.0286$

$P = 2.85$, $\Theta_R = 161$ No periodicity								
D	M	s_i	ϕ_i	Θ_i	$\Delta\Theta_i$	n_1	n_2	Q_B
26	10	295.33	0.44	158	3	1	2	0.0563
17	1	16.33	-0.46	-164	35	2	20	0.1105
25	8	234.33	0.04	13	148	3	91	0.4838

$P = 29.60$, $\Theta_R = -89$ No periodicity								
D	M	s_i	ϕ_i	Θ_i	$\Delta\Theta_i$	n_1	n_2	Q_B
25	8	234.33	-0.20	-73	17	1	13	0.3140
26	10	295.33	-0.14	-51	38	2	24	0.1492
17	1	16.33	0.43	156	115	3	67	0.2998
